# Enantiomeric Resolution and Docking Studies of Chiral Xanthonic Derivatives on Chirobiotic Columns

**DOI:** 10.3390/molecules23010142

**Published:** 2018-01-11

**Authors:** Ye‛ Zaw Phyo, Sara Cravo, Andreia Palmeira, Maria Elizabeth Tiritan, Anake Kijjoa, Madalena M. M. Pinto, Carla Fernandes

**Affiliations:** 1 ICBAS-Instituto de Ciências Biomédicas Abel Salazar, Universidade do Porto, Rua de Jorge Viterbo Ferreira, 228, 4050-313 Porto, Portugal; chemistkophyo.ckp@gmail.com (Y.Z.P.); ankijjoa@icbas.up.pt (A.K.); 2 Interdisciplinary Centre of Marine and Environmental Research (CIIMAR), Edifício do Terminal de Cruzeiros do Porto de Leixões, Av. General Norton de Matos s/n, 4050-208 Matosinhos, Portugal; scravo@ff.up.pt (S.C.); andreiapalmeira@gmail.com (A.P.); elizabeth.tiritan@iscsn.cespu.pt (M.E.T.); 3 Laboratório de Química Orgânica e Farmacêutica, Departamento de Ciências Químicas, Faculdade de Farmácia, Universidade do Porto, Rua de Jorge Viterbo Ferreira, 228, 4050-313 Porto, Portugal; 4 CESPU, Instituto de Investigação e Formação Avançada em Ciências e Tecnologias da Saúde (IINFACTS), Rua Central de Gandra, 1317, 4585-116 Gandra PRD, Portugal

**Keywords:** Chirobiotic^TM^, docking, enantioresolution, enantioselectivity, molecular recognition, xanthonic derivatives

## Abstract

A systematic study of enantioresolution of a library of xanthonic derivatives, prepared “in-house”, was successfully carried out with four commercially available macrocyclic glycopeptide-based columns, namely Chirobiotic^TM^ T, Chirobiotic^TM^ R, Chirobiotic^TM^ V and Chirobiotic^TM^ TAG. Evaluation was conducted in multimodal elution conditions: normal-phase, polar organic, polar ionic and reversed-phase. The effects of the mobile phase composition, the percentage of organic modifier, the pH of the mobile phase, the nature and concentration of different mobile phase additives on the chromatographic parameters are discussed. Chirobiotic^TM^ T and Chirobiotic^TM^ V, under normal-phase and reversed-phase modes, respectively, presented the best chromatographic parameters. Considering the importance of understanding the chiral recognition mechanisms associated with the chromatographic enantioresolution, and the scarce data available for macrocyclic glycopeptide-based columns, computational studies by molecular docking were also carried out.

## 1. Introduction

During the last few decades there has been widespread interest in oxygenated heterocyclic compounds such as molecules with a xanthone scaffold [1,2,3], mainly in consideration of their important roles as bioactive agents and because xanthone is an attractive core for molecular modifications [4,5,6,7,8,9] and the design of new molecular entities [10,11]. Xanthonic derivatives can be isolated from terrestrial [12,13] and marine [2] sources, or obtained by synthesis using different synthetic methodologies [14,15].

Recently, some synthetic enantiomerically pure xanthone derivatives prepared “in house” demonstrated highly interesting biological activities, namely inhibition of enzymes involved in inflammatory processes [16], growth inhibitory effects on different tumor cell lines [17], nerve sciatic conduction blockade [18], as well as the ability to behave as promising chiral selectors for liquid chromatography (LC) [19].

Since the enantiomeric purity of the final product is crucial regarding both biological activities and preparation of single enantiomer selectors for LC stationary phases, there is a great need for suitable methods for enantioseparation and evaluation of the enantiomeric composition. One of the most frequently applied techniques is LC with chiral columns [20]. Consequently, systematic studies of enantioresolution using different types of LC chiral columns, i.e., macrocyclic glycopeptide-based [21], Pirkle-type [22,23] and polysaccharide-based [24], and further determination of the enantiomeric purity of the synthesized xanthonic compounds, were described. By comparison, of the overall chromatographic results for the same small library of xanthonic derivatives considering the different types of chiral columns, in general, macrocyclic glycopeptide-based showed enantioselectivity but low resolution [25]. These results were not expected considering that this type of chiral columns are one of the most versatile and broadly applied, allowing efficient enantioseparations of diverse classes of chiral compounds [26,27,28,29,30,31,32,33]. Moreover, their usefulness can be increased considering the complementary profile in enantioselectivity of the different macrocyclic glycopeptide-based selectors [34].

In order to further explore the effectiveness of this type of columns to separate the enantiomers of this important class of compounds, herein we describe a systematic study of enantioresolution using a larger library of chiral xanthonic derivatives and different mobile phases in normal-phase mode (NPM), polar organic mode (POM), polar ionic mode (PIM), and reversed-phase mode (RPM). The macrocyclic glycopeptide columns based on ristocetin, teicoplanin, teicoplanin aglycone, and vancomycin, Chirobiotic^TM^ R, T, TAG and V, respectively, were evaluated. The effects of the mobile phase composition, the percentage of organic modifier, the pH of the mobile phase, the nature and concentration of different mobile phase additives on the chromatographic parameters, for each Chirobiotic^TM^ column, are discussed. The elution order was also assessed in all cases. Finally, taking into account the importance in understanding the chiral recognition mechanisms associated with the chromatographic enantioresolution [35,36] and the scarce data for macrocyclic glycopeptide-based selectors [37,38,39,40], computational studies were also carried out.

## 2. Results and Discussion

**Figure 1:**
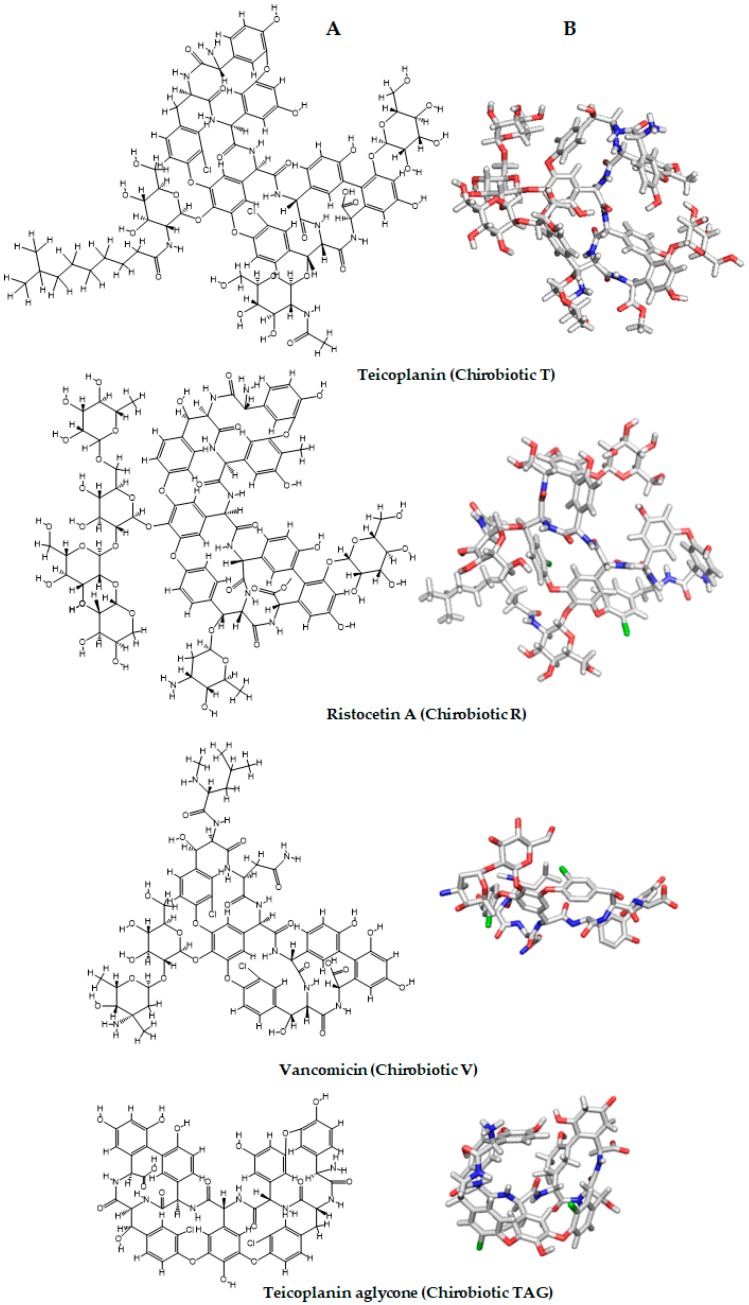
2D (**A**) and 3D (**B**) structures of the four macrocyclic glycopeptide-based selectors. Chiral selectors are represented in sticks with C, O, N, and Cl atoms colored in grey, red, blue and green, respectively.

**Figure 2:**
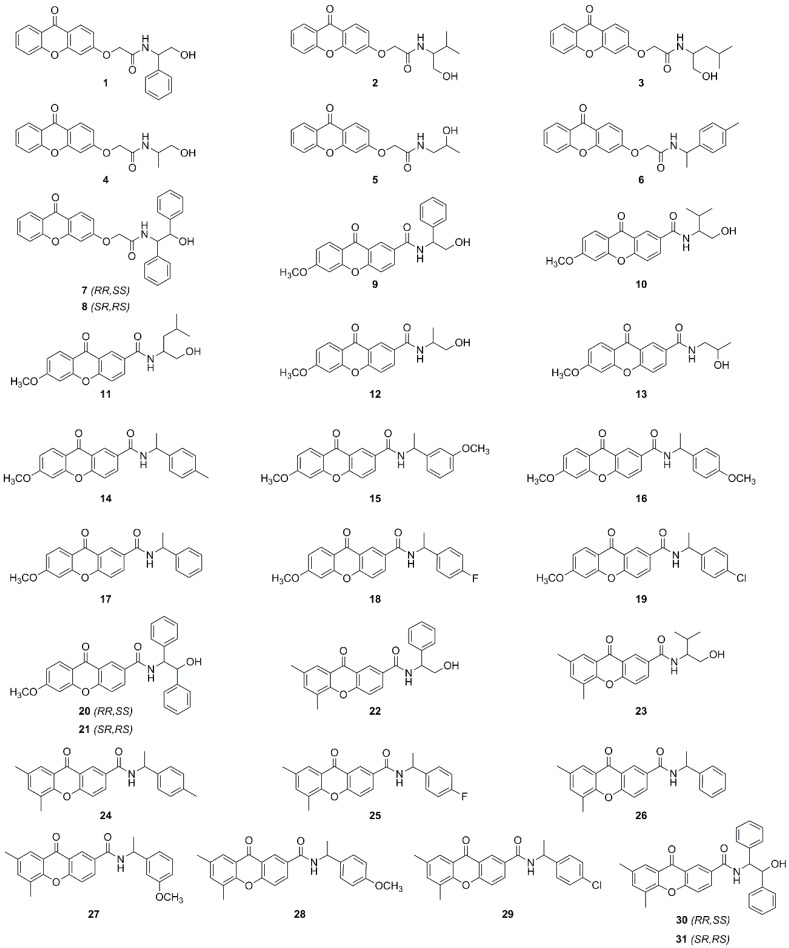
Chemical structures of chiral xanthonic analytes **1**–**31**.

In our previous work, LC columns containing macrocyclic glycopeptides as chiral selectors: Chirobiotic^TM^ R, T, TAG and V (Figure 1), were demonstrated to have enantioselectivity for six of the seven enantiomeric pairs of the xanthonic derivatives analyzed [21]. However, poor enantioresolution were obtained, which prompted us to pursue our investigation increasing the library of compounds and the variables of the mobile phase to better understand the behavior of these chiral columns towards the target analytes. In this work, a library of thirty-one chiral xanthonic analytes (Figure 2) was used for the systematic study of enantioresolution, including the previous seven enantiomeric pairs [21] and more twenty-four [22], applying the chiral columns based on these four macrocyclic glycopeptide selectors. 

**Scheme 1:**
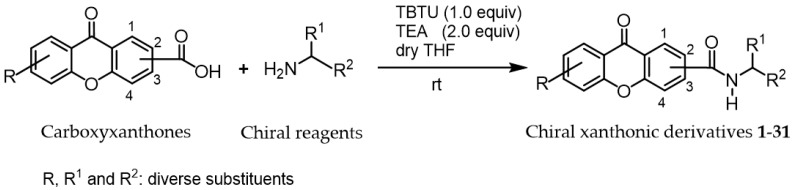
General scheme of synthesis of chiral xanthonic analytes **1**–**31**.

Chiral xanthonic analytes were obtained in enantiomeric pure form (enantiomeric excess between 98% and 99%) [21,22,24], by coupling carboxyxanthones with both enantiomers of commercially available chiral building blocks, using *O*-(benzotriazol-1-yl)-*N*,*N*,*N*′,*N*′-tetramethyluronium tetrafluoroborate (TBTU) as coupling reagent (Scheme 1), according to procedures described elsewhere [17,18]. 

The target library of this study, although comprising a common structural xanthone scaffold, is structurally diverse (Figure 2). The xanthone scaffold of analytes **1**–**8** has no substituents beyond an alkoxyamide chemical bridge for the link to the chiral moiety; analytes **9**–**21** have a methoxyl group at position 6 of the xanthone scaffold, and analytes **22**–**31** have methyl groups at positions 5 and 7. Additionally, the analytes **9**–**31** comprise an amide linkage with the chiral moiety at position 2. The majority of the analytes have only one stereogenic center with an alkyl or aromatic moiety, except **7**, **8**, **20**, **21**, **30** and **31** which have two stereogenic centers. 

Considering that chromatographic separations are highly influenced by mobile phase components, all chiral analytes were evaluated under four chromatographic elution modes (NPM, POM, PIM and RPM). In NPM, *n*-hexane (Hex) and an organic modifier, ethanol (EtOH) or 2-propanol (2-PrOH), were evaluated. Methanol (MeOH), EtOH, 2-PrOH or acetonitrile (ACN) in different proportions were used in POM. The mixture of MeOH with several percentages of triethylamine (TEA) and glacial acetic acid (AcOH) were employed in PIM. Regarding RPM, different proportions of MeOH or tetrahydrofuran (THF) and buffer (0.1 or 1% triethylammonium acetate (TEAA) or ammonium acetate (NH_4_OAc)) with pH 4.0–6.0 were applied.

The experimental conditions, including the nature and proportion of the organic modifier, the pH of the mobile phase, the buffer type and concentration, were investigated in the course of the enantioseparation process.

### 2.1. Performance of Chirobiotic T Column for Enantioresolution of Chiral Xanthonic Analytes

Table 1 summarizes the best chromatographic results obtained with Chirobiotic^TM^ T column, under multimodal elution conditions, with resolution factors ≥1.00. However, in some cases, results obtained with different mobile phases, but not leading to good enantioresolution, are also included for purposes of comparison.

Chirobiotic^TM^ T column presented good chromatographic parameters in our previous work [21], and in the present work the same pattern was achieved, with enantioseparation of twenty-five out of thirty-one enantiomeric mixtures of xanthonic analytes (81%) (Table 1).

In NPM, mixtures of Hex and EtOH or 2-PrOH as organic modifier, in different proportions, were evaluated in order to achieve optimized chromatographic conditions, i.e., high enantioselectivity and resolution in short analysis time. In general, the Hex:EtOH combinations afforded better chromatographic results. For example, excellent enantioselectivity (*α* = 2.09) and resolution (*R_S_* = 7.37) were obtained for analyte **17**, with a *k*_1_ value of 2.03, when Hex:EtOH (80:20, *v*/*v*) was used as mobile phase. This was also the best chromatographic result achieved for Chirobiotic^TM^ T column. Moreover, under NPM very high enantioselectivity and resolution were achieved for twenty-two analytes with *α* and *R_S_* values ranging from 1.11 to 2.09 and from 1.09 to 7.37, respectively. Additionally, it was found that, when using Hex:EtOH (90:10 *v*/*v*) as mobile phase, this column also showed enantioselectivity for analytes **24** and **29** but with slight resolution (*R_S_* < 1.00). On the other hand, POM using EtOH or 2-PrOH as mobile phases proved to be an excellent alternative to NPM for ten xanthonic analytes (Table 1), since good enantioselectivity and resolution were obtained (*α* and *R_S_* values ranging from 1.25 to 1.99 and from 1.00 to 1.90, respectively), with very short retention (values ranging from 0.30 to 1.63). As an example, high enantioselectivity (*α* = 1.49) and resolution (*R_S_* = 1.90) were obtained for analyte **10**, when 100% EtOH was used as mobile phase, being the chromatographic run less than 10 min. The low toxicity of this solvent is also a significant advantage to be taken into account [41,42].

Following the systematic investigation, PIM were also evaluated for the Chirobiotic^TM^ T column. In spite of the shorted retention factors obtained, absence or poor enantioselectivity was observed with all the combination of mobile phases evaluated. Only for analytes **4**, **9** and **20** *α* values > 1.00 were achieved but with poor resolution (*R_S_* < 1.00).

Similar to the achieved results in our previous work [21], high retention times were observed under RPM, however, for fourteen analytes good enantioselectivity and resolution were obtained, with *α* and *R_S_* values ranging from 1.16 to 2.83 and from 1.08 to 6.79, respectively. 

For example, the Chirobiotic^TM^ T column presented very high enantioseparation capabilities for analytes **15** and **23**, with *α* values of 1.33 and 2.83, respectively, and *R_S_* values of 2.46 and 6.79, respectively, using MeOH:TEAA at pH 4.2 (30:70 *v*/*v*) as mobile phase. These chromatographic results were also the best achieved for both analytes in this column. Moreover, the proportion 30:70 *v*/*v* of MeOH:TEAA was the optimum for the majority of the obtained enantioseparations.

**Figure 3:**
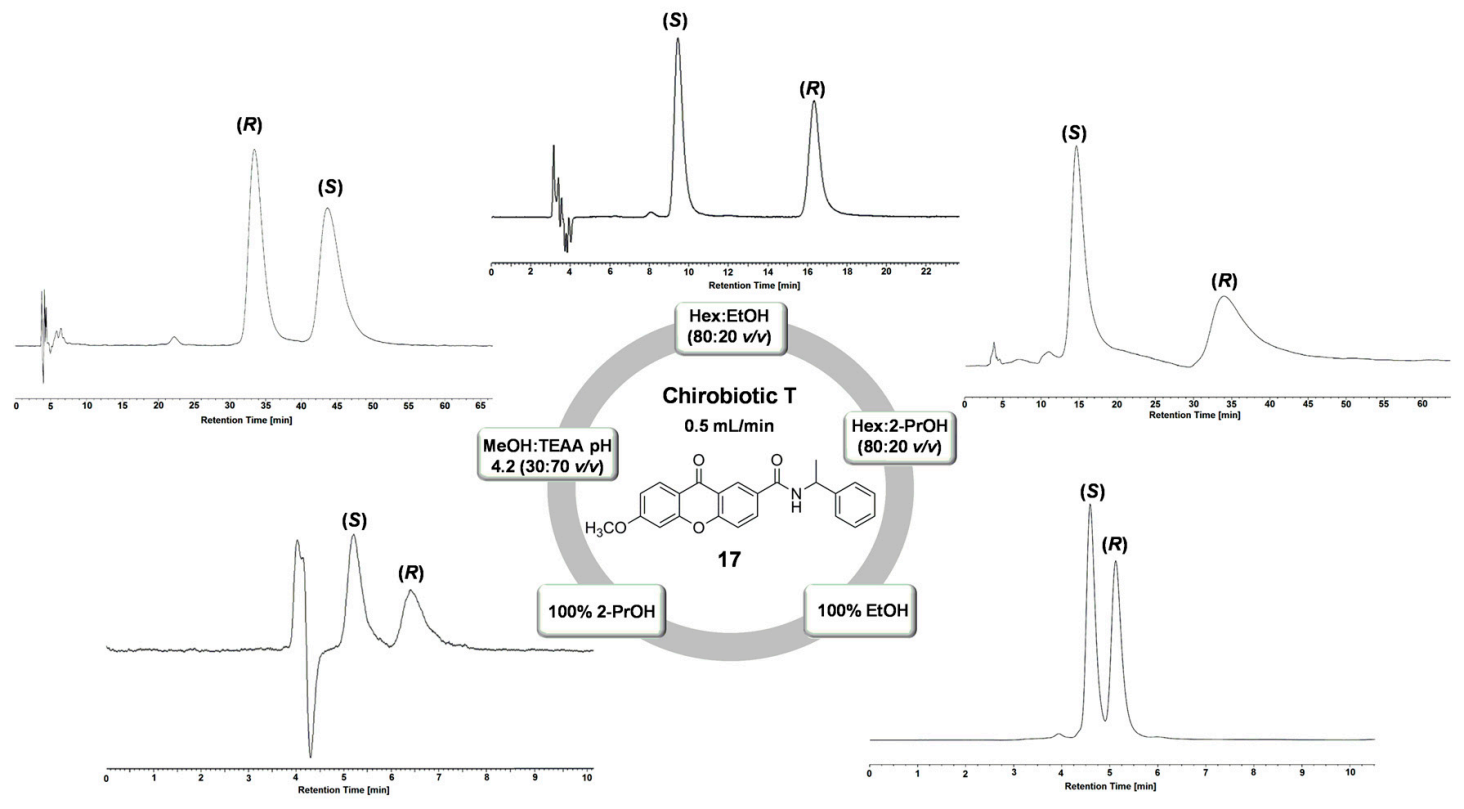
Chromatograms of the enantioseparation of analyte **17** on the Chirobiotic^TM^ T column using different mobile phases.

Considering the overall chromatographic data, it should be noted that analyte **17** could be baseline resolved under three different modes, namely NPM, POM and RPM. Figure 3 shows selected chromatograms of the enantioseparation of analyte **17** using different mobile phases.

Regarding the elution order, for the enantioseparated xanthonic analytes, the (*R*)-enantiomer was the first to elute in this column. The exception was only observed for analytes **17** and **23**, which elution order changed according to the elution mode: in NPM and POM the first eluted enantiomer was the (*S*), while (*R*) enantiomer eluted first in RPM. 

### 2.2. Performance of Chirobiotic^TM^ R Column for Enantioresolution of Chiral Xanthonic Analytes

Considering the Chirobiotic^TM^ R column, a total of seventeen chiral xanthonic analytes (55%) was enantioseparated with resolution factors ≥1.00 (Table 2). 

Under NPM, this column showed very high enantioseparation capabilities for nine analytes, with *α* and *R_S_* values ranging from 1.11 to 4.65 and from 1.12 to 3.95, respectively. The best chromatographic result was achieved for analyte **17**, with excellent enantioselectivity (*α* = 4.65), high resolution (*R_S_* = 2.05), and short retention time (*k*_1_ = 0.39), when Hex:2-PrOH (70:30, *v*/*v*) was used as mobile phase. Nevertheless, in general, mixtures of Hex:EtOH afforded better chromatographic results. 

It was found that none of the analytes which xanthone core has no substituents beyond an alkoxyamide chemical bridge for the link to the chiral moiety (**1**–**8**) were enantioseparated, under NPM. Actually, analytes **1**–**8** exhibited large *k* values and poor enantioresolution (data not shown). Consequently, under this elution mode, probably the nonspecific π-π interactions between the xanthonic and the aromatic moieties of the ristocetin selector resulted in high retention without chiral recognition. It is noteworthy that the nature of the substituents and their position on the xanthone core also have an important role in enantioselectivity.

Under POM, absence or poor resolution was observed for the majority of the xanthonic analytes. However, for the analytes comprising only one aromatic moiety and an hydroxyl group next to the stereogenic center (**1**, **9** and **22**) good enantioselectivity and resolution was obtained, with *α* and *R_S_* values ranging from 1.72 to 1.85 and from 1.00 to 1.32, respectively, using 100% MeOH or 2-PrOH as mobile phases. Therefore, these structural molecular features of the analytes might be determining for enantiorecognition under this elution conditions. Moreover, the nature of the alcoholic modifier also exerted considerable effects in the retention factors. The polar nature of the mobile phase increased in the sequence 2-PrOH, EtOH and MeOH, while at a constant percentage of alcohol modifier. Increasing carbon number of the molecules of the organic solvent is disadvantageous for polar interactions between the mobile phase and analytes and, consequently, the retention factor may increase [43]. This behavior was more pronounced for 2-PrOH. In this case, the steric effect probably contributes to the decreased interactions between the mobile phase and the analytes. As an example, the retention time of analyte **1** decreased when changing the mobile phase from 2-PrOH, EtOH and MeOH, with *k*_1_ values of 1.92, 0.56 and 0.27, respectively (data not shown).

Considering evaluation on the RPM the chromatographic results were only satisfactory for analytes **19**, **23**, **28** and **29**. The best enantioselectivity and resolution achieved were *α* = 1.70 and *R_S_* = 1.51 for analyte **23** using MeOH:TEAA pH 4.2 (40:60 *v*/*v*) as mobile phase. When replacing water by pure MeOH, i.e., changing RPM to PIM, interesting results were obtained for four analytes (**1**, **4**, **5** and **13**). The presence of the protic but less polar MeOH may improve the ion-dipole interactions between those analytes and the ristocetin selector. Moreover, the acidic and basic additives added to the mobile phases are important to ensure the ionization of the selector.

**Figure 4:**
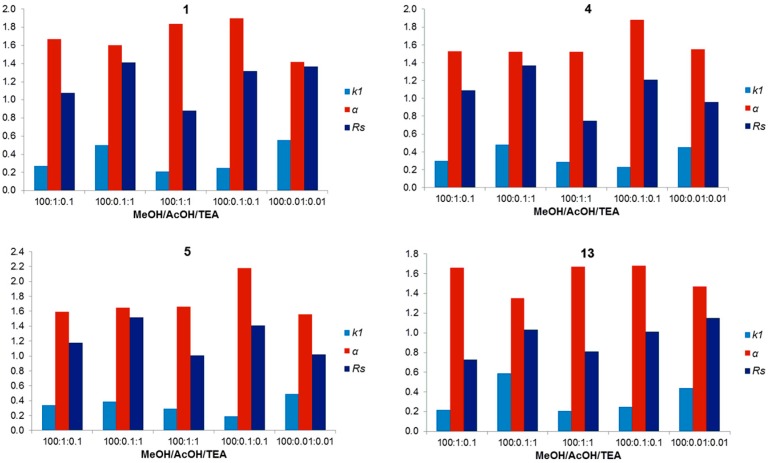
Effect of the different proportion of AcOH and TEA in MeOH on chromatographic parameters *k*_1_, *α* and *R_S_* for analytes **1**, **4**, **5** and **13**.

In order to investigate the effect of ion content on chromatographic parameters, experiments were carried out with mobile phases containing MeOH:AcOH:TEA in the ratio of 100:1:0.1, 100:0.1:1, 100:1:1, 100:0.1:0.1 and 100:0.01:0.01 *v*/*v*/*v*) (Figure 4). Generally, when using MeOH:AcOH:TEA 100:0.1:1 *v*/*v*/*v* high retention and resolution were obtained (*k*_1_ ranging from 0.39 to 0.59 and *R_S_* ranging from 1.03 to 1.52) with good enantioselectivity (*α* ranging from 1.35 to 1.65). Thus, using a slightly basic mobile phase, ion-dipole interactions between the amines of the selector and the amide or hydroxyl groups of the analytes, in addition to other selector-analyte interactions, may be responsible for chiral discrimination. Moreover, it was found that a higher ionic concentration resulted in lower retention. Probably, the ions existing in the mobile phase will compete for the interaction sites with the analytes.

In Chirobiotic^TM^ R column the elution order was also determined for all the enantioseparated xanthonic analytes. As shown in Table 2, for some analytes the first eluted enantiomer was the (*R*) while for others it was the (*S*). Accordingly, it was found that neither the xanthonic moiety of the analytes nor the chiral moiety determined the elution order. For example, comparing the results obtained with the analytes **19** and **29**, possessing the same chiral moiety but differing on the substituents linked to the xanthone scaffold, it can be seen that the first eluted enantiomer was the (*R*), while for the latter analyte was the (*S*)-enantiomer. Other situation can be observed when comparing the elution order of analytes **9** and **17**, possessing the same substituents linked to the xanthone scaffold but differing on the chiral moiety: for analyte **9** the first eluted enantiomer was the (*R*) while for analyte **17** was the (*S*)-enantiomer.

Moreover, in the case of analytes **10**, **13** and **23** the elution order changed according to the elution mode: in NPM and POM the first eluted enantiomer was the (*R*), differing for the opposite enantiomer in RPM (some data are not shown in Table 2 since it summarizes only the best chromatographic results obtained using Chirobiotic^TM^ R column for the tested xanthonic analytes).

### 2.3. Performance of Chirobiotic^TM^ V Column for Enantioresolution of Chiral Xanthonic Analytes

Considering the Chirobiotic^TM^ V column, NPM and RPM demonstrated to be the best elution modes for the enantioresolution of xanthonic analytes. A total of seven xanthonic analytes was successfully enantioseparated under NPM with *α* and *R_S_* values ranging from 1.13 to 2.31 and from 1.16 to 7.71, respectively. Considering RPM, very high enantioselectivity and resolution were obtained for fourteen analytes with *α* and *R_S_* values ranging from 1.21 to 3.32 and from 1.18 to 4.94, respectively. Relevant chromatographic results obtained using the Chirobiotic^TM^ V column for the target xanthonic analytes, under multimodal elution conditions are presented in Table 3.

Considering NPM, the combination of Hex and EtOH proved to be the most efficient. As an example, when Hex:EtOH (80:20 *v*/*v*) was used as mobile phase, excellent enantioselectivity (*α* = 2.31) and resolution (*R_S_* = 7.71) were obtained for analyte **17**, in a good retention time (*k*_1_ = 1.85). This is also the best chromatographic result achieved for all the chiral columns. 

The Chirobiotic^TM^ V column also afforded the best chromatographic results for other analytes (**6**, **14**, **15**, **16**, **18**, **19**, **24**, **25** and **29**) but under RPM. In this elution mode, the nature and proportion of the organic modifier, the pH of the mobile phase, the buffer type and concentration were investigated to study their influence on the enantioseparation process as well as to optimize the chromatographic parameters. It was found that the nature of the organic modifier exerted considerable effects on the separation, as example when MeOH was changed to THF the chromatographic parameters were not satisfactory (data not shown). Moreover, slight differences on enantioselectivity and resolution were observed at different pH (ranging from 4.0 to 6.0). This can be explained taking into account the neutral character of the analytes that, consequently, do not undergo much influence to variations of pH. The best mobile phases were the following: MeOH:NH_4_OAc pH 6 (50:50 *v*/*v*) and MeOH:NH_4_OAc pH 4 (50:50 *v*/*v*).

It was found that all the analytes having methyl groups at positions 5 and 7 of the xanthone scaffold and only one stereogenic center (**22**–**29**) were successfully enantioseparated under RPM. Opposite situation occurred for the analytes which xanthone scaffold has no substituents beyond an alkoxyamide chemical bridge for the link to the chiral moiety (**1**–**8**) (with exception of analyte **6**). Consequently, under this elution mode, the nature and position of the substituents on the xanthone scaffold also have a relevant influence on the chiral recognition. In the case of analytes **1**–**8**, probably the nonspecific hydrophobic interactions between the xanthone scaffold and the vancomycin selector resulted in retention without chiral recognition. 

Additionally, POM was found to be successful for the enantioseparation of two xanthonic analytes (**17** and **20**). The Chirobitic^TM^ V column provided excellent enantioselectivity (*α* = 2.00) and resolution (*R_S_* = 1.71) and very short time of analysis (*k*_1_ = 0.20) for analyte **17** with 100% 2-PrOH as mobile phase and, for analyte **20** the *α* and *R_S_* values were 1.36 and 1.23, respectively. Considering evaluation on PIM the chromatographic parameters were not satisfactory (data not shown).

Regarding the elution order, likewise to the Chirobiotic^TM^ T column, the (*R*)-enantiomer of all the enantioseparated xanthonic analytes was the first to elute in Chirobiotic^TM^ V column, except for analytes **9** and **17**, which elution order changed according to the elution mode: in NPM and POM the first eluted enantiomer was (*S*), while in RPM was the (*R*)-enantiomer.

**Figure 5:**
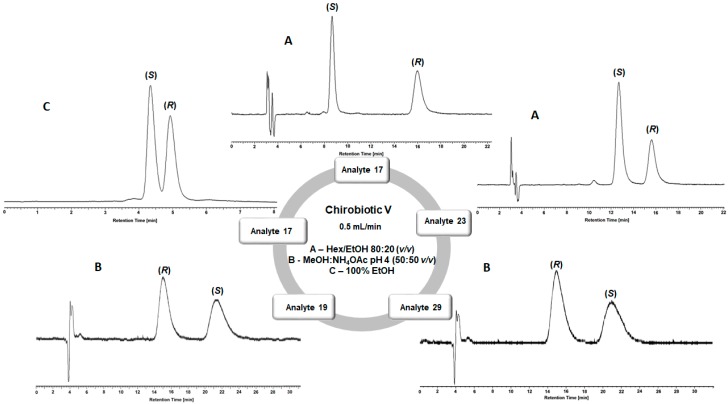
Chromatograms of xanthonic analytes **17**, **19**, **23** and **29** on the Chirobiotic^TM^ V column.

Selected chromatograms for the separation of chiral xanthonic analytes on the Chirobiotic^TM^ V column are shown in Figure 5.

### 2.4. Performance of Chirobiotic^TM^ TAG Column for Enantioresolution of Chiral Xanthonic Analytes

The performance of Chirobiotic^TM^ TAG column to resolve the xanthonic analytes was also systematically evaluated under multimodal elution conditions. However, this column showed much lower discrimination capability compared to the others. Table 4 showed the relevant chromatographic results obtained for the tested xanthonic analytes, under multimodal elution conditions.

As shown in Table 4, good enantioseparation was observed under NPM for five xanthonic analytes using Hex:EtOH (70:30 *v*/*v*) as mobile phase with *α* and *R_S_* values ranging from 1.37 to 1.45 and from 1.02 to 3.87, respectively. Partial enantioseparation was obtained for analytes **1** and **4**, under POM, using 100% EtOH as mobile phase.

Considering elution order of all the enantioseparated xanthonic analytes, the (*R*)-enantiomer was the first to elute on the Chirobiotic^TM^ TAG column. The only exception was also observed for analyte **17**.

### 2.5. Overall Effectiveness of Chirobiotic^TM^ Columns for Enantioresolution of Chiral Xanthonic Compounds under Multimodal Elution Conditions

**Figure 6:**
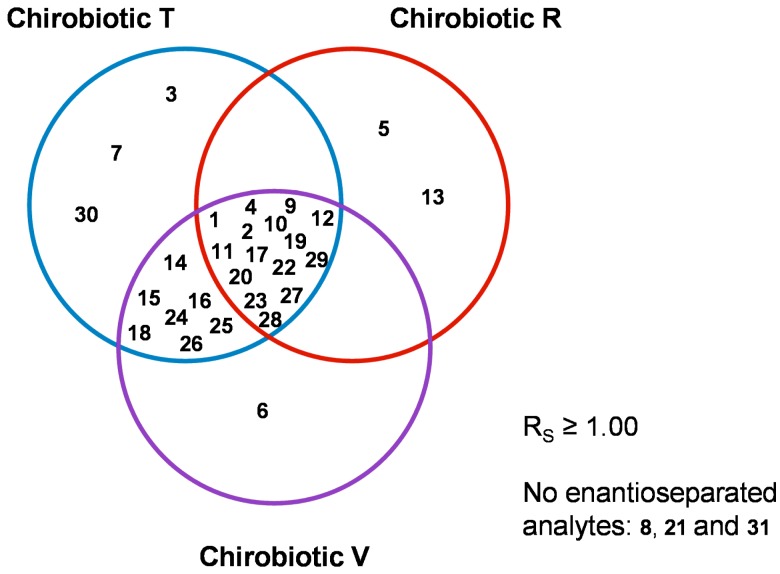
Diagram considering the xanthonic analytes enantioseparated by Chirobiotic^TM^ T, R and V columns, with resolution factors ≥1.00. Chirobiotic^TM^ TAG was not included considering its poor enantioselectivity for the evaluted analytes.

After the systematic study of enantioseparation by LC under multimodal elution conditions, Chirobiotic^TM^ T demonstrated to be the most suitable for the xanthonic analytes evaluated. In fact, twenty-five out of thirty-one xanthonic analytes (81%) were enantioseparated with *R_S_* values ≥ 1.00 (Figure 6).

**Figure 7:**
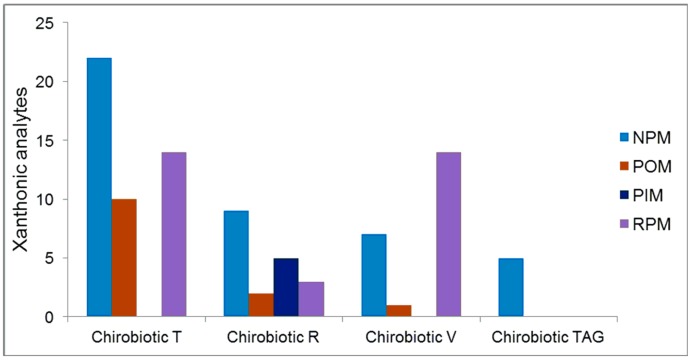
Comparative performance of Chirobiotic^TM^ T, R, V and TAG for baseline enantioseparation of xanthonic analytes under multimodal elution conditions.

Moreover, this column was more efficient under NPM, allowing the efficient enantioseparation of twenty-two analytes (71%), as shown in Figure 7. This result is in agreement with our previous work [21]. Similar trend was observed by Armstrong *et col.* for dihydrofurocoumarins [44]. RPM and POM were also efficient elution modes for the efficient enantioseparation of fourteen (45%) and ten xanthonic analytes (32%), respectively, on a Chirobiotic^TM^ T column. Moreover, in some cases these elution modes proved to be good alternatives to NPM. Additionally, it should be highlight that analytes **3**, **7** and **30** could only be enantioresolved with *R_S_* value ≥ 1.00 on the Chirobiotic^TM^ T column. 

Interestingly, Chirobiotic^TM^ V under RPM was very efficient for the tested xanthonic analytes (Figure 7) and, in some cases, exhibited the broadest enantioselectivity observed in all chiral columns. It was a surprising result considering that in our previous work, this chiral column was one of the less efficient. However, herein, for the new xanthonic analytes evaluated, particularly those having methyl groups at positions 5 and 7 on the xanthone scaffold, this column was very effective. In fact, a total of twenty-three analytes (74%) were baseline enantioseparated on the Chirobiotic^TM^ V column (Figure 6), and analyte **6** could only be baseline resolved in this column.

Chirobiotic^TM^ R and TAG columns showed the lowest discrimination ability, under multimodal elution conditions. However, under NPM nine (29%) and five (16%) analytes were baseline enantioseparated in Chirobiotic^TM^ R and TAG, respectively (Figure 7). Additionally, Chirobiotic^TM^ R column was the only one useful for the efficient enantioseparation of analytes **5** and **13** (Figure 6).

Finally, it should be emphasized that the xanthonic analytes comprising two stereogenic centers with the same configuration, i.e., analytes **8**, **21** and **31**, (Figure 2) were not enantioseparated in any of the four Chirobiotic^TM^ columns. Interestingly, their diastereoisomers were enantioseparated. These results suggest that the stereochemistry of both stereogenic centers on the xanthonic analytes play an important role in the enantioseparation of this class of chiral compounds on macrocyclic glycopeptide-based columns evaluated.

### 2.6. Computational Studies

In order to understand the binding mechanism behind the observed results of enantioresolution, docking studies were performed, using the chiral xanthonic analytes that were enantioseparated by LC with resolution factors ≥1.00. Values of docking scores are presented on Table 5. The lower the docking score, the more stable is the analyte:selector complex. Concerning Chirobiotic^TM^ T, R, V, and TAG columns, there was a 52%, 50%, 47%, and 80% agreement between docking scores and experimental results concerning the elution order of the enantiomers of the chiral analytes. The differences observed may be due to the complex structures of the macrocyclic glycopeptide-based selectors allowing different binding patterns with the molecules of the analytes. In fact, although all the structurally related glycopeptide antibiotics can establish the same type of interactions, including hydrogen, dipole-dipole, π-stacking, hydrophobic as well as steric repulsion [20], their morphological differences in the aglycon macrocyclic portions as well as other structural features, are responsible for their differences in enantioselectivity, retention times, and efficiency [45]. Moreover, computationally, AutodockVina has a hybrid scoring function (empirical and knowledge-based function) inspired in the X-Score function that consists of a weighted sum of steric interactions, hydrophobic interaction, and number of active rotatable bonds with different weights [46]. Therefore, similar selectors and/or ligands can originate very different docking scores, depending not only on the type of interactions established, but also the number of interactions, maximizing favorable and minimizing unfavorable interactions, shape and property complementarities [47]. 

**Figure 8:**
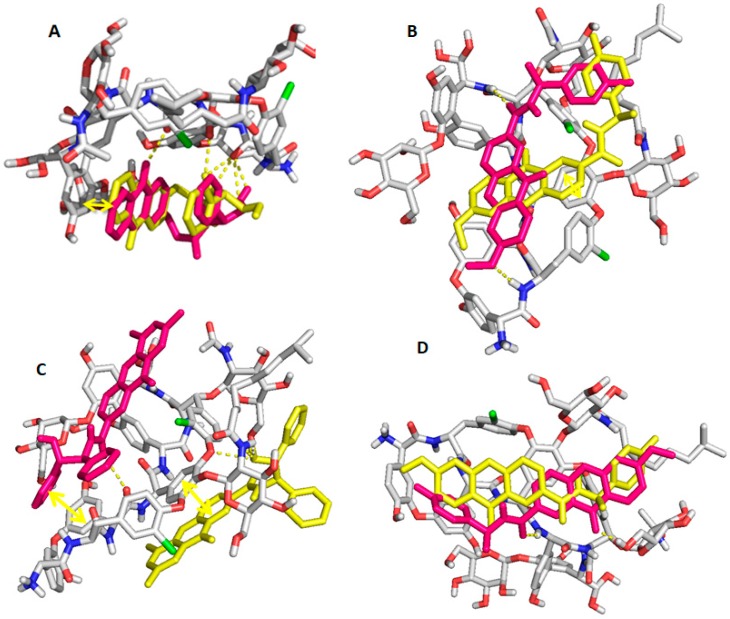
Analyte **1** (**A**), Analyte **14** (**B**), Analyte **30** (**C**), and Analyte **18** (**D**), docked on teicoplanin selector. Chiral selectors are represented in sticks with C, O, N, and Cl atoms colored in grey, red, blue, and green, respectively. (*S*) and (*R*) enantiomers are represented with magenta and yellow sticks, respectively. Hydrogen interactions and π-stacking interactions are represented with dashes and double arrow, respectively.

**Figure 9:**
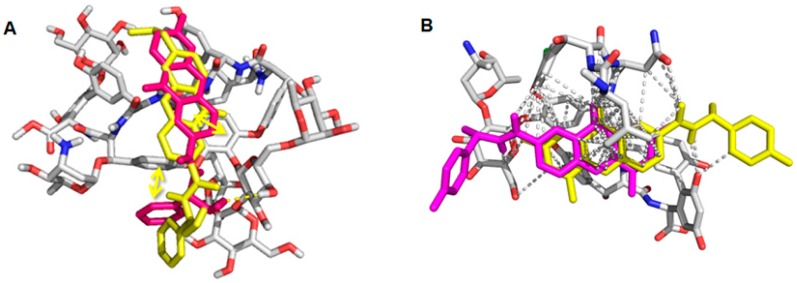
(**A**) Analyte **9** docked on ristocetin selector; (**B**) Analyte **25** docked on vancomycin selector. Chiral selectors are represented in sticks with C, O, N, and Cl atoms colored in grey, red, blue, and green, respectively. (*S*) and (*R*) enantiomers are represented with magenta and yellow sticks, respectively. In (**A**), hydrogen interactions and π-stacking interactions are represented with dashes and double arrow, respectively. In (**B**), all the interactions (apolar and polar) are represented as grey dashes.

**Figure 10:**
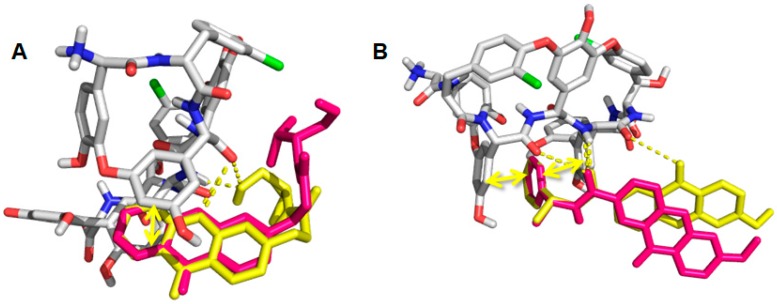
(**A**) Analyte **2** docked on teicoplanin aglycone selector; (**B**) Analyte **17** docked on teicoplanin aglycone selector. Chiral selectors are represented in sticks with C, O, N, and Cl atoms colored in grey, red, blue, and green, respectively. (*S*) and (*R*) enantiomers are represented with magenta and yellow sticks, respectively. Hydrogen interactions and π-stacking interactions are represented with dashes and double arrow, respectively.

In order to understand the binding mechanism, a visual inspection of the binding conformations and established interactions were performed for the molecules of the xanthonic analytes. Figure 8, Figure 9 and Figure 10 illustrate representative examples of the most stable docked conformations for enantiomers complexes with the chiral selectors of the four chiral columns.

Figure 8A show that both enantiomers of analyte **1** are folded in a “U” shape to maximize both the π stacking interactions between the xanthonic moiety and one of the teicoplanin aromatic rings; and the hydrogen interactions between –OH and –NH– groups of the xanthone molecule and the teicoplanin carbonyl groups. However, as the carbonyl group of the xanthone core of (*S*)-enantiomer is turned inside of the teicoplanin molecule, an additional hydrogen interaction with a teicoplanin carbonyl group was established. This last interaction can justify the stronger retention of (*S*)-enantiomer in the Chirobiotic^TM^ T column.

However, this is not a general rule for the other enantioseparated analytes. As shown in Figure 8, for analytes **14** (Figure 8B), **30** (Figure 8C), and **18** (Figure 8D), the docking poses of both enantiomers of each xanthonic pair are very diverse. Consequently, different groups of both xanthonic and chiral moieties of the analytes are involved in the interactions. 

Although both enantiomers of analyte **9** bind through π-stacking interactions between the xanthone scaffold and the aromatic rings of the ristocetin selector, only the aromatic ring of the chiral moiety of (*S*)-enantiomer is in such a way that allows an additional parallel π-stacking interaction (Figure 9A). Moreover, the terminal –OH group of (*S*)-enantiomer also establishes an additional hydrogen interaction with an –OH group of the chiral selector.

On the Chirobiotic^TM^ V column, the majority of the chiral xanthonic analytes were enantioseparated under RPM. Concerning the enantiomers of analyte **25** docked onto the Chirobiotic^TM^ V column, both enantiomers are bound to the selector through hydrophobic interactions between the xanthone scaffold and the aromatic rings and alkyl chain of vancomycin selector (Figure 9B). Additionally, hydrogen interactions (three) were also established. However, one of the hydrogen interactions on (*S*)-enantiomer is established with a fluorine atom [48], more electronegative than oxygen, thus resulting on a stronger interaction.

On the Chirobiotic^TM^ TAG column, the (*S*)-enantiomers were always more retained, with exception of analyte **17**. Using analyte **2** as an example, both enantiomers are bound to the Chirobiotic^TM^ TAG column by π stacking interactions established between the xanthone scaffold and aromatic rings of the selector. (*S*)-enantiomer binds more stably to the chiral selector creating additional hydrogen interactions with the amide groups of the chiral moiety (Figure 10A). Concerning analyte **17**, the aromatic ring and amide group of the chiral moiety on both enantiomers establish π-stacking and hydrogen interactions with the chiral selector, respectively. Nonetheless, only the (*R*)-enantiomer establishes an extra hydrogen interaction through the xanthone carbonyl group (Figure 10B). This different elution profile of analyte **17**, comparing to the other analytes, was due to its binding pose with the xanthone scaffold facing the outside medium, and the aromatic ring of the chiral moiety establishing double sided π-stacking interactions with two aromatic rings on the selector (Figure 10B); whereas for other analytes, such as **2**, the xanthone scaffold is the structural moiety that is holding the analyte to the selector through parallel π-stacking interactions (Figure 10A).

## 3. Materials and Methods

### 3.1. Chemicals and Reagents

Chiral xanthonic analytes (Figure 2) were obtained “in-house” according to procedures described elsewhere [17,18]. HPLC grade EtOH, MeOH, 2-PrOH, ACN, THF, and Hex were purchased from Sigma-Aldrich (St. Louis, MO, USA). TEA, AcOH, NH_4_OAc, all p.a. grade, were also obtained from Sigma-Aldrich. Ultrapure water was purified by a Milli-Q system (Millipore, Bedford, MA, USA). TEAA buffers were prepared by titration of 0.1 or 1% (by volume) aqueous solutions of TEA with AcOH to adjust to a suitable pH.

### 3.2. Apparatus and Chromatography

Chromatographic measurements were carried out on two chromatographic systems. One LC system contained a model 880-PU Intelligent HPLC pump (JASCO Corporation, Tokyo, Japan), equipped with a 7125 injector (Rheodyne LCC, Rohnert Park, CA, USA) fitted with a 20 µL loop, a JASCO model 880-30 solvent mixer, a 875-UV intelligent UV/Vis detector A chromatography station for Microsoft Windows 95, version 1.7 DLL, was applied. The second LC was a Dionex Ultimate 3000 (Thermo Fisher Scientific Inc., Waltham, MA, USA) system equipped with a 3000 quaternary pump, a 3000 autosampler, and a 3000 Variable UV/Vis detector was also used. Chromeleon^TM^ software version 7.2 Ultimate (Thermo Fisher Scientific Inc., Waltham, MA, USA) was employed to manage chromatographic data. Chromatographic separations were performed on four commercial macrocyclic glycopeptide-based columns: Chirobiotic^TM^ T (150 × 4.6 mm id, 5 µm particle size), Chirobiotic^TM^ TAG (150 × 2.1 mm id, 5 µm particle size), Chirobiotic^TM^ V (250 × 4.6 mm id, 5 µm particle size) and Chirobiotic^TM^ R (150 × 2.1 mm id, 5 µm particle size) (Figure 1), from ASTEC (Whippany, NJ, USA). Stock solutions of all chiral xanthonic analytes were prepared by dissolution in EtOH at a concentration of 1 mg/mL. Working solutions were further prepared by dilution of the stock solutions in the same solvent to a concentration of 10 µg/mL. The injection volume was 10 μL, and all the chromatographic analyses were performed in isocratic mode at 22 ± 2 °C, in triplicate. The dead times (t_0_) were considered to be equal to the peak of the solvent front, and were taken from each particular run. The column flow rate was 0.5 mL/min (Chirobiotic^TM^ T and V) or 0.2 mL/min (Chirobiotic^TM^ R and TAG), and the chromatograms were recorded by UV detection at a wavelength of 254 nm. The mobile phases were prepared in a volume/volume relation, filtered using a Millipore 0.45 µm filter, and further degassed in an ultrasonic bath for 15 min before use. All the analytes mentioned in Figure 2 were evaluated with different mobile phases under NPM, POM, PIM and RPM. The elution order was determined for all the enantioseparated xanthonic analytes by injecting the solutions of the enantiomeric mixtures (prepared mixing equal aliquots of each enantiomer), and then each enantiomer separately.

### 3.3. Computational

The structures of the selectors were obtained from PubChem [49], and both enantiomers of all xanthonic compounds (Figure 2) were drawn and minimized using an Austin Model 1 (AM1) semi-empirical quantum mechanics force field [50]. The calculation was finished when the gradient between any two successive steps in the geometry search was less than 10^−1^ kcal/mol/Å or the maximum steps were reached, whichever comes first. The line search used was the Broyden-Fletcher-Golfarb-Shanno search which uses an approximate Hessian matrix to guide the search [51]. Docking simulations between the chiral selector and the xanthonic enantiomers were undertaken in AutoDock Vina (Molecular Graphics Lab, La Jolla, CA, USA) [46]. AutoDock Vina considered the target conformation as a rigid unit while the ligands were allowed to be flexible and adaptable to the target. Vina searched for the lowest binding affinity conformations and returned nine different conformations for each small molecule. The lowest binding energy docking poses of each compound were chosen. AutoDock Vina was run using an exhaustiveness of 9 and grid boxes engulfing the selectors were built. PyMol v1.3 (Schrödinger, New York, NY, USA) [52] was used for visual inspection of results and graphical representations.

## 4. Conclusions

The systematic study of enantioseparation for thirty-one chiral xanthonic analytes using four macrocyclic glycopeptide-based chiral columns confirmed the applicability of these columns in multimodal elution conditions for the enantioseparation of this class of compounds. It was found that their applicability increased considering the complementary profile in enantioselectivity of the different macrocyclic glycopeptide-based selectors. For some chiral xanthonic analytes, 100% EtOH as mobile phase presented excellent chromatographic parameters. EtOH is considered a green solvent due its low toxicity. As it was expected, the structural nature of the analytes and the chiral selectors as well as the mobile phase composition proved to be fundamental factors for the molecular interactions and consequently for chiral recognition.

The docking study showed that each macrocyclic glycopeptide-based selector allowed different binding patterns for both enantiomers of the analytes due to their complex structures. Due to the very diverse structural features of the thirty-one enantiomeric pairs of xanthonic analytes, with different alkyl and aryl substituents in different positions of the xanthone scaffold, and to the differences in spatial disposition (chirality) and tridimensional shape of the compounds, would not be suitable to establish a generalized chiral recognition mechanism or identify which chemical characteristics are more relevant for retention in the referred columns.

However, the results of the present study fulfilled the initial objectives since higher resolutions were obtained for the chiral xanthonic analytes evaluated compared to our previous work, and additional information about chiral recognition mechanisms was provided. In summary, we believe that this work contributes to a better knowledge in enantioseparation and chiral recognition in general and for chiral xanthonic derivatives in particular. Computational chemistry studies allowed an improvement to understand the behavior of these important class of compounds within macrocyclic glycopeptide-based chiral columns.

## Figures and Tables

**Table 1:** Chromatographic data, retention factor of first eluted enantiomer (*k*_1_), separation factor (*α*), resolution (*R_S_*), and elution order, for the chiral xanthonic analytes on Chirobiotic^TM^ T column, under multimodal chromatographic conditions.

Analyte	Elution Mode	Mobile Phase	*k*_1_	*α*	*R_S_*	First Eluted Enantiomer
**1**	NPM	Hex:EtOH (80:20 *v*/*v*)	18.13	1.16	2.26	(*R*)
	NPM	Hex:2-PrOH (70:30 *v*/*v*)	33.19	1.47	1.93	(*R*)
	POM	100% EtOH	1.19	1.14	0.70	(*R*)
**2**	NPM	Hex:EtOH (40:60 *v*/*v*)	1.92	1.26	1.28	(*R*)
	NPM	Hex:2-PrOH (70:30 *v*/*v*)	15.98	1.62	1.49	(*R*)
	POM	100% EtOH	0.80	1.20	0.86	(*R*)
**3**	NPM	Hex:EtOH (80:20 *v*/*v*)	32.34	1.09	1.09	(*R*)
	NPM	Hex:2-PrOH (50:50 *v*/*v*)	2.93	1.32	1.00	(*R*)
**4**	NPM	Hex:EtOH (80:20 *v*/*v*)	19.56	1.42	2.95	(*R*)
	PIM	MeOH:AcOH:TEA (100:0.1:1 *v*/*v*/*v*)	1.13	1.09	0.80	(*R*)
	POM	100% EtOH	1.09	1.33	1.28	(*R*)
**7**	NPM	Hex:EtOH (90:10 *v*/*v*)	23.91	1.29	2.83	(*R*)
**9**	NPM	Hex:EtOH (70:30 *v*/*v*)	9.21	1.47	2.48	(*R*)
	NPM	Hex:2-PrOH (50:50 *v*/*v*)	7.11	1.46	1.07	(*R*)
	PIM	MeOH:AcOH:TEA (100:0.1:0.1 *v*/*v*/*v*)	1.34	1.08	0.60	(*R*)
	POM	100% EtOH	1.13	1.42	1.73	(*R*)
	POM	100% 2-PrOH	1.63	1.63	1.32	(*R*)
**10**	NPM	Hex:EtOH (70:30 *v*/*v*)	5.04	1.54	3.30	(*R*)
	NPM	Hex:2-PrOH (50:50 *v*/*v*)	4.05	1.61	1.56	(*R*)
	POM	100% EtOH	0.80	1.49	1.90	(*R*)
	POM	100% 2-PrOH	1.01	1.74	1.61	(*R*)
**11**	NPM	Hex:EtOH (80:20 *v*/*v*)	6.93	1.29	2.43	(*R*)
	NPM	Hex:2-PrOH (80:20 *v*/*v*)	8.35	1.33	0.84	(*R*)
	POM	100% EtOH	0.63	1.25	1.00	(*R*)
	POM	100% 2-PrOH	0.69	1.41	1.14	(*R*)
**12**	NPM	Hex:EtOH (80:20 *v*/*v*)	15.31	1.32	2.31	(*R*)
	NPM	Hex:2-PrOH (70:30 *v*/*v*)	22.72	1.38	0.50	(*R*)
	POM	100% EtOH	1.03	1.25	1.00	(*R*)
**14**	NPM	Hex:EtOH (90:10 *v*/*v*)	9.78	1.29	1.55	(*R*)
	NPM	Hex:2-PrOH (50:50 *v*/*v*)	1.53	1.15	0.78	(*R*)
	RPM	MeOH:TEAA pH 4.2 (40:60 *v*/*v*)	10.54	1.19	1.53	(*R*)
**15**	NPM	Hex:EtOH (90:10 *v*/*v*)	16.67	1.26	1.97	(*R*)
	NPM	Hex:2-PrOH (50:50 *v*/*v*)	2.33	1.23	0.89	(*R*)
	POM	100% EtOH	0.67	1.15	0.70	(*R*)
	RPM	MeOH:TEAA pH 4.2 (30:70 *v*/*v*)	20.59	1.33	2.46	(*R*)
**16**	NPM	Hex:EtOH (90:10 *v*/*v*)	17.86	1.18	1.51	(*R*)
	NPM	Hex:2-PrOH (50:50 *v*/*v*)	2.48	1.15	0.40	(*R*)
	POM	100% EtOH	0.70	1.08	0.50	(*R*)
	RPM	MeOH:TEAA pH 4.2 (30:70 *v*/*v*)	22.32	1.24	1.92	(*R*)
**17** *	NPM	Hex:EtOH (80:20 *v*/*v*)	2.03	2.09	7.37	(*S*)
	NPM	Hex:2-PrOH (80:20 *v*/*v*)	3.33	2.71	3.31	(*S*)
	POM	100% EtOH	0.42	1.39	1.45	(*S*)
	POM	100% 2-PrOH	0.30	1.99	1.78	(*S*)
	RPM	MeOH:TEAA pH 4.2 (30:70 *v*/*v*)	21.13	1.49	3.20	(*R*)
**18**	NPM	Hex:EtOH (90:10 *v*/*v*)	12.05	1.16	1.43	(*R*)
	NPM	Hex:2-PrOH (50:50 *v*/*v*)	1.67	1.13	0.30	(*R*)
	RPM	MeOH:TEAA pH 4.2 (30:70 *v*/*v*)	18.89	1.18	1.37	(*R*)
**19**	NPM	Hex:EtOH (90:10 *v*/*v*)	10.68	1.13	1.28	(*R*)
	NPM	Hex:2-PrOH (50:50 *v*/*v*)	1.59	1.10	0.30	(*R*)
	RPM	MeOH:TEAA pH 4.2 (30:70 *v*/*v*)	29.61	1.17	1.30	(*R*)
**20**	NPM	Hex:EtOH (80:20 *v*/*v*)	11.20	1.31	2.24	(*R*)
	NPM	Hex:2-PrOH (50:50 *v*/*v*)	3.45	1.30	1.11	(*R*)
	PIM	MeOH:AcOH:TEA (100:0.1:1 *v*/*v*/*v*)	1.10	1.11	0.74	(*R*)
	POM	100% EtOH	0.85	1.30	1.32	(*R*)
	POM	100% 2-PrOH	1.09	1.46	0.99	(*R*)
	RPM	MeOH:TEAA pH 4.2 (30:70 *v*/*v*)	8.55	2.26	1.66	(*R*)
**22**	NPM	Hex:EtOH (80:20 *v*/*v*)	5.50	1.36	2.45	(*R*)
	NPM	Hex:2-PrOH (50:50 *v*/*v*)	2.62	1.39	1.18	(*R*)
	POM	100% EtOH	0.72	1.34	1.38	(*R*)
	POM	100% 2-PrOH	0.77	1.52	1.10	(*R*)
**23** *	NPM	Hex:EtOH (50:50 *v*/*v*)	1.22	1.25	1.28	(*S*)
	NPM	Hex:2-PrOH (50:50 *v*/*v*)	1.57	1.19	0.87	(*S*)
	POM	100% EtOH	0.56	1.25	1.00	(*S*)
	POM	100% 2-PrOH	0.48	1.54	1.08	(*S*)
	RPM	MeOH:TEAA pH 4.2 (40:60 *v*/*v*)	4.68	2.83	6.79	(*R*)
**24**	NPM	Hex:EtOH (90:10 *v*/*v*)	3.57	1.11	0.79	(*R*)
	RPM	MeOH:TEAA pH 4.2 (30:70 *v*/*v*)	29.11	1.23	1.55	(*R*)
**25**	NPM	Hex:EtOH (90:10 *v*/*v*)	4.09	1.09	0.92	(*R*)
	RPM	MeOH:TEAA pH 4.2 (30:70 *v*/*v*)	22.38	1.16	1.08	(*R*)
**26**	NPM	Hex:EtOH (90:10 *v*/*v*)	4.53	1.12	1.16	(*R*)
	RPM	MeOH:TEAA pH 4.2 (30:70 *v*/*v*)	21.58	1.22	1.49	(*R*)
**27**	NPM	Hex:EtOH (90:10 *v*/*v*)	5.91	1.16	1.40	(*R*)
	RPM	MeOH:TEAA pH 4.2 (40:60 *v*/*v*)	10.87	1.26	1.97	(*R*)
**28**	NPM	Hex:EtOH (90:10 *v*/*v*)	6.41	1.11	1.13	(*R*)
	RPM	MeOH:TEAA pH 4.2 (30:70 *v*/*v*)	27.13	1.26	1.87	(*R*)
**29**	NPM	Hex:EtOH (90:10 *v*/*v*)	4.67	1.07	0.89	(*R*)
	RPM	MeOH:TEAA pH 4.2 (40:60 *v*/*v*)	13.04	1.16	1.18	(*R*)
**30**	NPM	Hex:EtOH (90:10 *v*/*v*)	15.16	1.24	1.93	(*R*)
	NPM	Hex:2-PrOH (80:20 *v*/*v*)	9.76	1.33	0.88	(*R*)
	POM	100% EtOH	0.56	1.17	0.70	(*R*)
	POM	100% 2-PrOH	0.49	1.43	1.05	(*R*)

Chromatographic conditions: flow rate: 0.5 mL/min, UV detection at 254 nm; * Elution order changed with the mobile phase.

**Table 2:** Chromatographic data, retention factor of first eluted enantiomer (*k*_1_), separation factor (*α*), resolution (*R_S_*), and elution order, for the chiral xanthonic analytes on Chirobiotic^TM^ R column, under multimodal chromatographic conditions.

Analyte	Elution Mode	Mobile Phase	*k*_1_	*α*	*R_S_*	First Eluted Enantiomer
**1**	POM	100% MeOH	0.27	1.72	1.23	(*S*)
	PIM	MeOH:AcOH:TEA (100:0.1:1 *v*/*v*/*v*)	0.50	1.60	1.41	(*S*)
**2**	POM	100% MeOH	0.27	1.44	0.45	(*R*)
	PIM	MeOH:AcOH:TEA (100:0.01:0.01 *v*/*v*/*v*)	0.48	1.32	0.81	(*R*)
**4**	POM	100% MeOH	0.23	1.88	1.00	(*S*)
	PIM	MeOH:AcOH:TEA (100:0.1:1 *v*/*v*/*v*)	0.48	1.52	1.37	(*S*)
**5**	POM	100% MeOH	0.28	1.80	1.02	(*R*)
	PIM	MeOH:AcOH:TEA (100:0.1:1 *v*/*v*/*v*)	0.39	1.65	1.52	(*R*)
**9**	NPM	Hex:EtOH (80:20 *v*/*v*)	9.32	1.46	1.99	(*R*)
	NPM	Hex:2-PrOH (70:30 *v*/*v*)	7.59	1.88	1.49	(*R*)
	POM	100% 2-PrOH	1.44	1.85	1.32	(*R*)
	PIM	MeOH:AcOH:TEA (100:0.1:0.1 *v*/*v*/*v*)	0.22	1.72	0.87	(*R*)
**10** *	NPM	Hex:EtOH (90:10 *v*/*v*)	15.78	1.24	1.77	(*R*)
	NPM	Hex:2-PrOH (80:20 *v*/*v*)	8.41	1.69	1.73	(*R*)
**11**	NPM	Hex:EtOH (90:10 *v*/*v*)	10.83	1.16	1.68	(*R*)
**12**	NPM	Hex:EtOH (90:10 *v*/*v*)	28.07	1.16	1.37	(*R*)
	PIM	MeOH:AcOH:TEA (100:0.1:1 *v*/*v*/*v*)	0.67	1.26	0.84	(*R*)
**13** *	PIM	MeOH:AcOH:TEA (100:0.01:0.01 *v*/*v*/*v*)	0.44	1.47	1.15	(*S*)
**17**	NPM	Hex:EtOH (90:10 *v*/*v*)	2.32	2.91	3.95	(*S*)
	NPM	Hex:2-PrOH (70:30 *v*/*v*)	0.39	4.65	2.05	(*S*)
	POM	100% 2-PrOH	0.29	2.05	0.91	(*S*)
	RPM	MeOH:TEAA pH 4.2 (50:50 *v*/*v*)	1.09	1.35	0.70	(*S*)
**19**	RPM	MeOH:TEAA pH 4.2 (30:70 *v*/*v*)	1.90	1.21	1.00	(*R*)
**20**	NPM	Hex:EtOH (90:10 *v*/*v*)	20.33	1.11	1.18	(*R*)
	NPM	Hex:2-PrOH (50:50 *v*/*v*)	2.13	1.28	0.91	(*R*)
	PIM	MeOH:AcOH:TEA (100:0.1:1 *v*/*v*/*v*)	1.10	1.11	0.74	(*R*)
**22**	NPM	Hex:EtOH (90:10 *v*/*v*)	9.69	1.58	2.81	(*R*)
	NPM	Hex:2-PrOH (70:30 *v*/*v*)	2.06	2.21	1.83	(*R*)
	POM	100% 2-PrOH	0.61	1.97	1.00	(*R*)
**23** *	NPM	Hex:2-PrOH (80:20 *v*/*v*)	2.42	1.52	1.12	(*R*)
	POM	100% 2-PrOH	0.41	1.36	0.50	(*R*)
	RPM	MeOH:TEAA pH 4.2 (40:60 *v*/*v*)	0.64	1.70	1.51	(*S*)
**27**	RPM	MeOH:TEAA pH 4.2 (30:70 *v*/*v*)	15.54	1.09	0.70	(*S*)
**28**	NPM	Hex:EtOH (90:10 *v*/*v*)	6.41	1.11	1.13	(*S*)
	RPM	MeOH:TEAA pH 4.2 (30:70 *v*/*v*)	15.45	1.25	1.12	(*S*)
**29**	RPM	MeOH:TEAA pH 4.2 (40:60 *v*/*v*)	13.04	1.16	1.18	(*S*)

Chromatographic conditions: flow rate: 0.2 mL/min, UV detection at 254 nm; * Elution order changed with the mobile phase.

**Table 3:** Chromatographic data, retention factor of first eluted enantiomer (*k*_1_), separation factor (*α*), resolution (*R_S_*), and elution order, for the chiral xanthonic analytes on the Chirobiotic^TM^ V column, under multimodal chromatographic conditions.

Analyte	Elution Mode	Mobile Phase	*k*_1_	*α*	*R_S_*	First Eluted Enantiomer
**1**	RPM	MeOH:NH_4_OAc pH 6 (50:50 *v*/*v*)	2.32	1.12	0.92	(*R*)
**2**	NPM	Hex:EtOH (80:20 *v*/*v*)	8.72	1.09	0.88	(*R*)
**4**	NPM	Hex:EtOH (50:50 *v*/*v*)	3.37	1.08	0.66	(*R*)
**6**	NPM	Hex:EtOH (80:20 *v*/*v*)	4.69	1.09	0.89	(*R*)
	RPM	MeOH:NH_4_OAc pH 6 (50:50 *v*/*v*)	3.21	1.39	2.29	(*R*)
	RPM	MeOH:NH_4_OAc pH 4 (50:50 *v*/*v*)	2.68	1.39	2.67	(*R*)
**9** *	NPM	Hex:EtOH (80:20 *v*/*v*)	15.72	1.19	1.16	(*S*)
	POM	100% EtOH	0.72	1.17	0.90	(*S*)
**10**	NPM	Hex:EtOH (80:20 *v*/*v*)	8.45	1.24	1.89	(*R*)
	POM	100% EtOH	0.39	1.23	0.60	(*R*)
	POM	100% 2-PrOH	1.24	1.29	0.60	(*R*)
**11**	NPM	Hex:EtOH (80:20 *v*/*v*)	6.14	1.15	1.39	(*R*)
**12**	NPM	Hex:EtOH (80:20 *v*/*v*)	13.56	1.13	1.35	(*R*)
	POM	100% 2-PrOH	1.79	1.11	0.60	(*R*)
**14**	RPM	MeOH:NH_4_OAc pH 6 (50:50 *v*/*v*)	3.52	1.43	1.56	(*R*)
	RPM	MeOH:NH_4_OAc pH 4 (50:50 *v*/*v*)	2.98	1.47	2.24	(*R*)
**15**	RPM	MeOH:NH_4_OAc pH 6 (50:50 *v*/*v*)	3.41	1.42	2.32	(*R*)
	RPM	MeOH:NH_4_OAc pH 4 (50:50 *v*/*v*)	2.87	1.44	2.49	(*R*)
**16**	RPM	MeOH:NH_4_OAc pH 6 (50:50 *v*/*v*)	3.49	1.35	2.08	(*R*)
	RPM	MeOH:NH_4_OAc pH 4 (50:50 *v*/*v*)	2.98	1.36	1.85	(*R*)
**17** *	NPM	Hex:EtOH (80:20 *v*/*v*)	1.85	2.31	7.71	(*S*)
	POM	100% EtOH	0.13	2.20	1.29	(*S*)
	POM	100% 2-PrOH	0.20	2.00	1.71	(*S*)
	RPM	MeOH:NH_4_OAc pH 6 (50:50 *v*/*v*)	3.11	1.21	1.18	(*R*)
	RPM	MeOH:NH_4_OAc pH 4 (50:50 *v*/*v*)	2.61	1.21	1.05	(*R*)
**18**	RPM	MeOH:NH_4_OAc pH 6 (50:50 *v*/*v*)	4.26	1.04	0.90	(*R*)
	RPM	MeOH:NH_4_OAc pH 4 (50:50 *v*/*v*)	2.66	1.37	1.89	(*R*)
**19**	RPM	MeOH:NH_4_OAc pH 6 (50:50 *v*/*v*)	4.09	1.53	2.79	(*R*)
	RPM	MeOH:NH_4_OAc pH 4 (50:50 *v*/*v*)	3.03	1.55	2.47	(*R*)
**20**	NPM	Hex:EtOH (80:20 *v*/*v*)	3.39	1.21	1.32	(*R*)
	POM	100% EtOH	0.51	1.25	0.60	(*R*)
	POM	100% 2-PrOH	1.77	1.36	1.23	(*R*)
**22**	RPM	MeOH:NH_4_OAc pH 6 (50:50 *v*/*v*)	2.38	1.13	0.75	(*R*)
	RPM	MeOH:NH_4_OAc pH 4 (50:50 *v*/*v*)	2.51	1.15	0.78	(*R*)
**23**	NPM	Hex:EtOH (80:20 *v*/*v*)	3.45	1.31	2.68	(*R*)
	RPM	MeOH:NH_4_OAc pH 6 (50:50 *v*/*v*)	1.34	2.34	4.94	(*R*)
	RPM	MeOH:NH_4_OAc pH 4 (50:50 *v*/*v*)	1.35	3.32	4.65	(*R*)
**24**	RPM	MeOH:NH_4_OAc pH 6 (50:50 *v*/*v*)	3.39	1.43	1.17	(*R*)
	RPM	MeOH:NH_4_OAc pH 4 (50:50 *v*/*v*)	3.17	1.32	1.80	(*R*)
**25**	RPM	MeOH:NH_4_OAc pH 6 (50:50 *v*/*v*)	3.00	1.10	1.45	(*R*)
	RPM	MeOH:NH_4_OAc pH 4 (50:50 *v*/*v*)	2.55	1.35	1.66	(*R*)
**26**	RPM	MeOH:NH_4_OAc pH 6 (50:50 *v*/*v*)	2.94	1.27	1.32	(*R*)
	RPM	MeOH:NH_4_OAc pH 4 (50:50 *v*/*v*)	2.47	1.28	1.39	(*R*)
**27**	RPM	MeOH:NH_4_OAc pH 6 (50:50 *v*/*v*)	3.14	1.38	1.91	(*R*)
	RPM	MeOH:NH_4_OAc pH 4 (50:50 *v*/*v*)	2.29	1.41	1.79	(*R*)
**28**	RPM	MeOH:NH_4_OAc pH 6 (50:50 *v*/*v*)	2.88	1.32	1.28	(*R*)
	RPM	MeOH:NH_4_OAc pH 4 (50:50 *v*/*v*)	2.39	1.34	1.50	(*R*)
**29**	RPM	MeOH:NH_4_OAc pH 6 (50:50 *v*/*v*)	3.31	1.49	1.97	(*R*)
	RPM	MeOH:NH_4_OAc pH 4 (50:50 *v*/*v*)	2.99	1.54	2.08	(*R*)

Chromatographic conditions: flow rate: 0.5 mL/min, UV detection at 254 nm; * Elution order changed with the mobile phase.

**Table 4:** Chromatographic data, retention factor of first eluted enantiomer (*k*_1_), separation factor (*α*), resolution (*R_S_*), and elution order, for the chiral xanthonic analytes on the Chirobiotic^TM^ TAG column, under multimodal chromatographic conditions.

Analyte	Elution Mode	Mobile Phase	*k*_1_	*α*	*R_S_*	First Eluted Enantiomer
**1**	POM	100% EtOH	1.26	1.24	0.60	(*R*)
**2**	NPM	Hex:EtOH (70:30 *v*/*v*)	5.01	1.42	1.45	(*R*)
	POM	100% EtOH	0.73	1.39	0.80	(*R*)
**4**	NPM	Hex:EtOH (70:30 *v*/*v*)	10.66	1.38	1.76	(*R*)
	POM	100% EtOH	1.21	1.36	0.92	(*R*)
**5**	NPM	Hex:EtOH (70:30 *v*/*v*)	8.29	1.37	1.02	(*R*)
**17**	NPM	Hex:EtOH (70:30 *v*/*v*)	0.80	2.77	3.87	(*S*)
**30**	NPM	Hex:EtOH (70:30 *v*/*v*)	2.01	1.45	1.16	(*R*)
**31**	NPM	Hex:EtOH (70:30 *v*/*v*)	1.57	1.27	0.50	(*R*)

Chromatographic conditions: flow rate: 0.2 mL/min, UV detection at 254 nm.

**Table 5:** Docking scores (Kcal.mol^−1^) of both enantiomers of xanthonic analytes on Chirobiotic^TM^ T, R, V, TAG (**I**), and binding affinity difference between (*R*) and (*S*) enantiomers (**II**).

					I				II			
Analytes	*R_S_* Value				Docking Score				Docking Score (*R*)–(*S*) (Delta) δ			
	T	R	V	TAG	T	R	V	TAG	T	R	V	TAG
(*R*)-**1** (*S*)-**1**	2.26	1.41	<1.00	<1.00	**−6.6** **−7.1**	**−7.1** **−6.9**	*	*	−0.5	−0.2	*	*
(*R*)-**2** (*S*)-**2**	1.49	<1.00	<1.00	1.45	−5.9 −5.4	*	*	**−4.9** **−5.1**	−0.5	*	*	−0.2
(*R*)-**3** (*S*)-**3**	1.09	<1.00	<1.00	<1.00	−5.8 −5.8	*	*	*	0.0	*	*	*
(*R*)-**4** (*S*)-**4**	2.95	1.37	<1.00	1.76	**−5.8** **−5.9**	**−6.2** **−6.0**	*	−4.9 −4.8	−0.1	−0.2	*	−0.1
(*R*)-**5** (*S*)-**5**	<1.00	1.52	<1.00	1.02	*	−6.3 −6.1	*	**−5.1** **−5.2**	*	−0.2	*	−0.1
(*R*)-**6** (*S*)-**6**	<1.00	<1.00	2.67	<1.00	*	*	−6.9 −6.6	*	*	*	−0.3	*
(*R*,*R*)-**7** (*S*,*S*)-**7**	2.83	<1.00	<1.00	<1.00	−7.0 −6.7	*	*	*	−0.3	*	*	*
(*S*,*R*)-**8** (*R*,*S*)-**8**	<1.00	<1.00	<1.00	<1.00	*	*	*	*	*	*	*	*
(*R*)-**9** (*S*)-**9**	2.48	1.99	1.16	<1.00	−6.2 −6.1	**−6.2** **−6.7**	**	*	−0.1	−0.5	**	*
(*R*)-**10** (*S*)-**10**	3.30	1.77	1.89	<1.00	−5.7 −5.7	**	−6.7 −6.6	*	0.0	**	−0.1	*
(*R*)-**11** (*S*)-**11**	2.43	1.68	1.39	<1.00	**−5.7** **−5.8**	−6.3 −6.1	**−6.1** **−6.3**	*	−0.1	−0.2	−0.2	*
(*R*)-**12** (*S*)-**12**	2.31	1.37	1.35	<1.00	−5.9 −5.8	**−6.2** **−6.5**	−6.7 −6.5	*	−0.1	−0.3	−0.2	*
(*R*)-**13** (*S*)-**13**	<1.00	1.15	<1.00	<1.00	*	**	*	*	*	**	*	*
(*R*)-**14** (*S*)-**14**	1.55	<1.00	2.24	<1.00	**−6.5** **−6.6**	*	**−6.5** **−6.6**	*	−0.1	*	−0.1	*
(*R*)-**15** (*S*)-**15**	2.46	<1.00	2.49	<1.00	−6.3 −6.3	*	−7.3 −7.1	*	0.0	*	−0.2	*
(*R*)-**16** (*S*)-**16**	1.92	<1.00	2.08	<1.00	−6.0 −5.9	*	**−7.1** **−7.4**	*	−0.2	*	−0.3	*
(*R*)-**17** (*S*)-**17**	7.37	3.95	7.71	3.87	**	−7.3 −7.3	**	**−4.9** **−4.6**	**	0.0	**	−0.3
(*R*)-**18** (*S*)-**18**	1.43	<1.00	1.89	<1.00	**−5.6** **−5.7**	*	**−7.7** **−7.9**	*	−0.1	*	−0.2	*
(*R*)-**19** (*S*)-**19**	1.30	1.00	2.79	<1.00	−6.5 −6.4	**−7.2** **−7.3**	−7.7 −7.5	*	−0.1	−0.1	−0.2	*
(*R*,*R*)-**20** (*S*,*S*)-**20**	2.24	1.18	1.32	<1.00	−6.5 −6.5	−7.4 −7.1	**−7.6** **−7.8**	*	0.0	−0.3	−0.2	*
(*S*,*R*)-**21** (*R*,*S*)-**21**	<1.00	<1.00	<1.00	<1.00	*	*	*	*	*	*	*	*
(*R*)-**22** (*S*)-**22**	2.45	2.81	<1.00	<1.00	−6.5 −6.2	−7.1 −7.0	*	*	−0.3	−0.1	*	*
(*R*)-**23** (*S*)-**23**	6.79	1.51	4.94	<1.00	**	**	−6.3 −6.7	*	**	**	−0.4	*
(*R*)-**24** (*S*)-**24**	1.55	<1.00	1.80	<1.00	**−6.7** **−7.2**	*	−8.0 −7.5	*	−0.5	*	−0.5	*
(*R*)-**25** (*S*)-**25**	1.08	<1.00	1.66	<1.00	**−6.1** **−6.7**	*	**−7.9** **−8.3**	*	−0.6	*	−0.4	*
(*R*)-**26** (*S*)-**26**	1.49	<1.00	1.39	<1.00	**−6.5** **−7.2**	*	**−7.6** **−7.8**	*	−0.5	*	−0.2	*
(*R*)-**27** (*S*)-**27**	1.97	<1.00	1.91	<1.00	**−7.0** **−7.1**	*	**−7.5** **−7.6**	*	−0.1	*	−0.1	*
(*R*)-**28** (*S*)-**28**	1.87	1.13	1.50	<1.00	**−6.3** **−6.8**	**−7.5** **−7.2**	−7.8 −7.5	*	−0.5	−0.3	−0.3	*
(*R*)-**29** (*S*)-**29**	1.18	1.18	2.08	<1.00	**−6.6** **−7.1**	−7.6 −7.6	−7.7 −7.5	*	−0.5	0.0	−0.2	*
(*R*,*R*)-**30** (*S*,*S*)-**30**	1.93	<1.00	<1.00	1.16	**−6.6** **−6.8**	*	*	**−5.4** **−5.5**	−0.2	*	*	−0.1
(*S*,*R*)-**31** (*R*,*S*)-**31**	<1.00	<1.00	<1.00	<1.00	*	*	*	*	*	*	*	*

* not analyzed because *R_S_* < 1.00; ** not analyzed because elution order was different according to the mobile phase used; **Bold numbers**: docking binding affinity in accordance with elution order.

## Supplementary Materials

Supplementary File 1
