# Supplementary material for: Enantiomeric Resolution and Docking Studies of Chiral Xanthonic Derivatives on Chirobiotic Columns
Source: Molecules. 2018 Jan 11;23(1):142. doi: 10.3390/molecules23010142 (PMC6017832; doi:10.3390/molecules23010142)
Supplement: Supplementary File 1 [file molecules-23-00142-s001.pdf]

Supplementary Material:

Analyte **1**
Hex:2-PrOH (70:30 *v*/*v*)

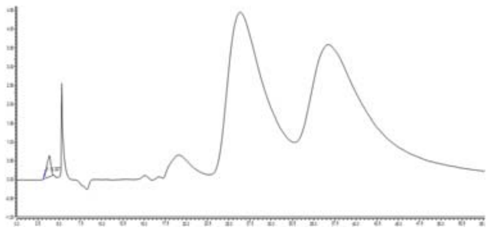

Analyte **2**
Hex:EtOH (40:60 *v*/*v*)

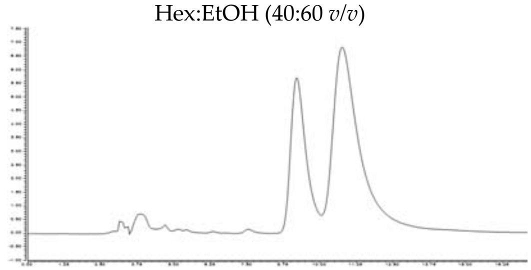

Analyte **2**
100 % EtOH

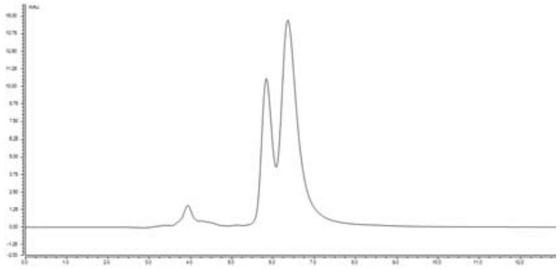

Analyte **3**
Hex:2-PrOH (50:50 *v*/*v*)

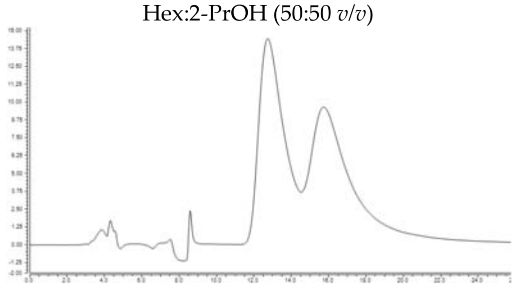

Analyte **4**
Hex:EtOH (80:20 *v*/*v*)

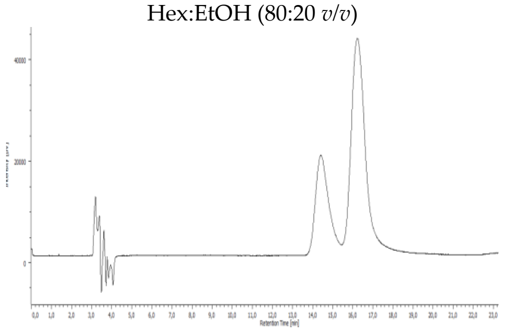

Analyte **9**
100 % EtOH

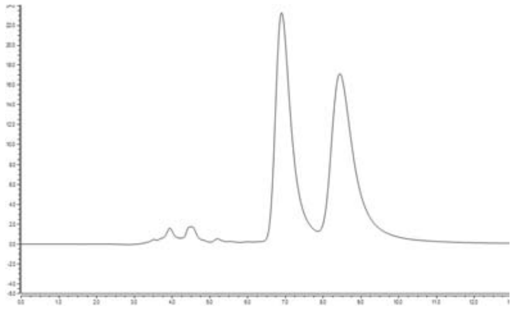

Analyte **10**
Hex:EtOH (70:30 *v*/*v*)

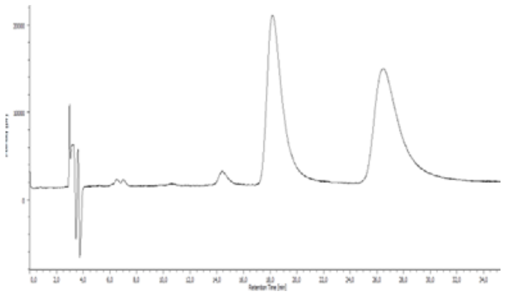

Analyte **10**
Hex:2-PrOH (50:50 *v*/*v*)

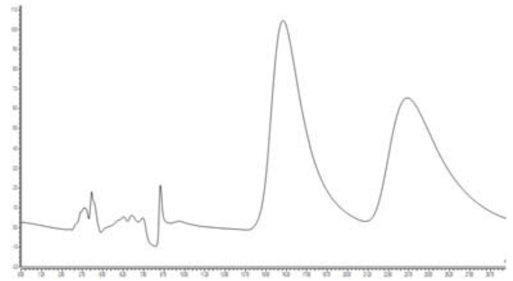

SFigure 1: Chromatograms for the enantioseparation of analytes **1-4,** **9** and **10** on Chirobiotic T column using different mobile phases. Chromatographic conditions: Flow rate: 0.5 mL/min, UV detection at 254 nm.

Analyte **10**
100 % EtOH

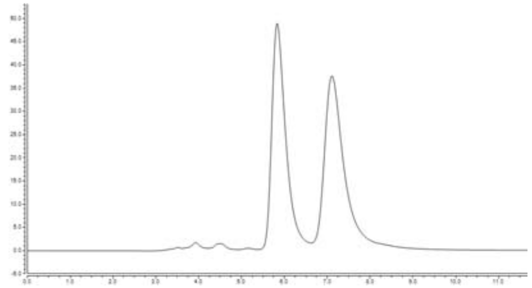

Analyte **10**
100 % 2-PrOH

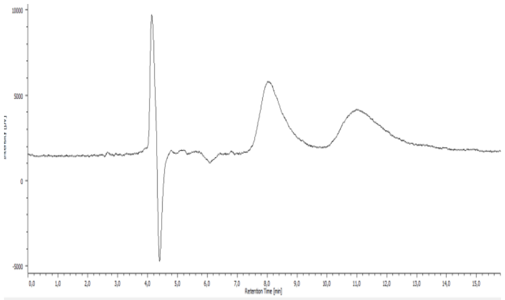

Analyte **11**
Hex:EtOH (80:20 *v/v*)

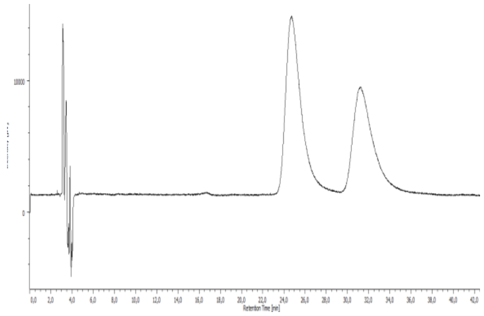

Analyte **11**
100 % EtOH

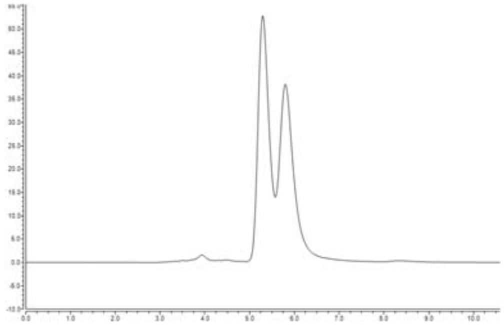

Analyte **12**
Hex:EtOH (80:20 *v/v)*

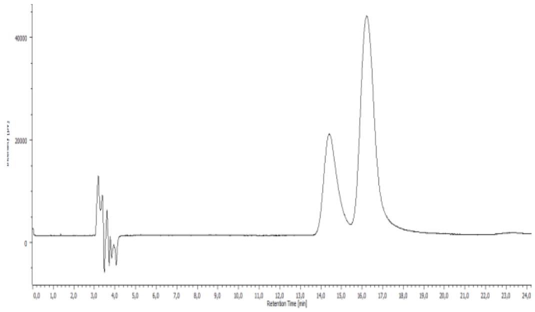

Analyte **14**
Hex:EtOH (90:10 *v/v)*

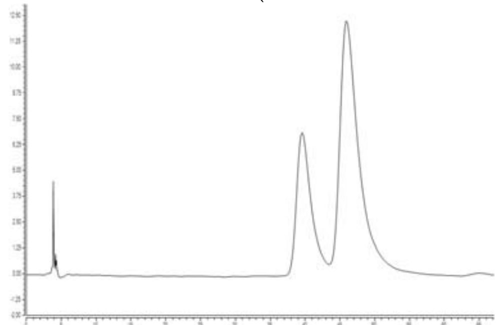

Analyte **14**
Hex:2-PrOH (50:50 *v/v*)

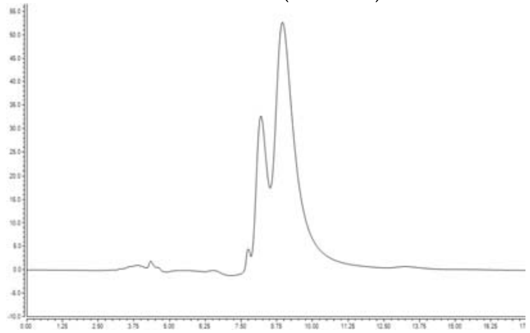

Analyte **14**
MeOH:TEAA pH 4.2 (40:60 *v/v)*

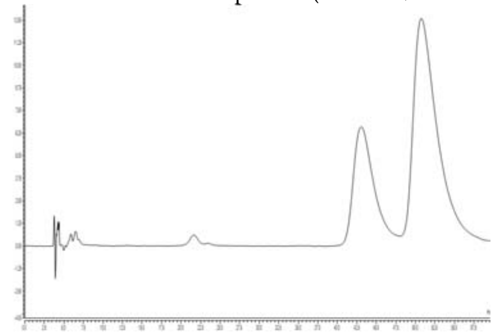

SFigure 2: Chromatograms for the enantioseparation of analytes **10-12** and **14** on Chirobiotic T column using different mobile phases. Chromatographic conditions: Flow rate: 0.5 mL/min, UV detection at 254 nm.

Analyte **15**
Hex:EtOH (90:10 *v/v)*

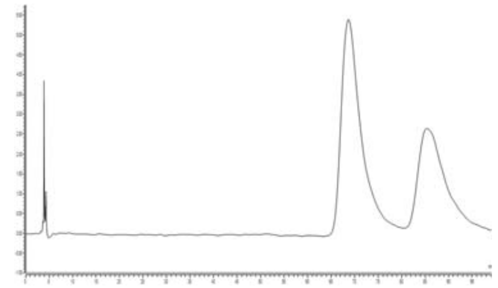

Analyte **15**
Hex:2-PrOH (50:50 *v*/*v*)

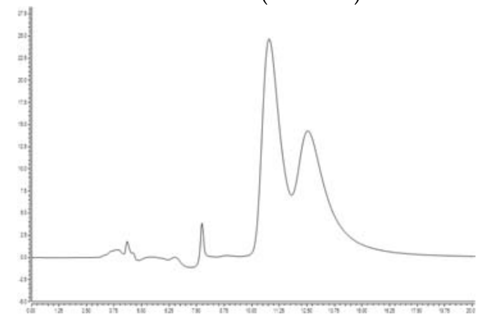

Analyte **16**
Hex:2-PrOH (50:50 *v*/*v*)

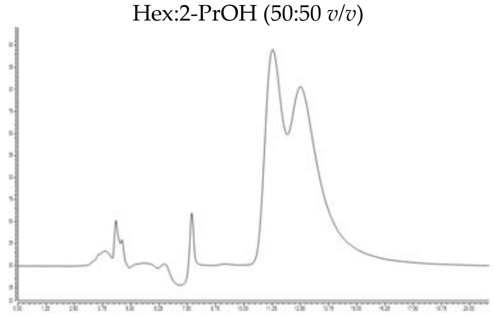

Analyte **17**
Hex:EtOH (80:20 *v/v)*

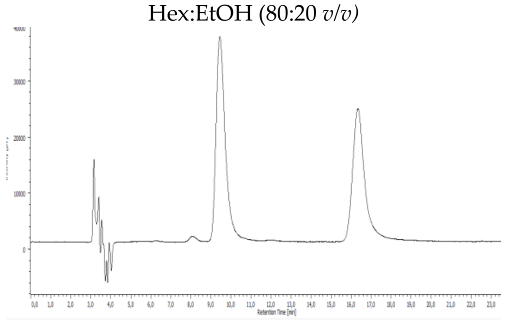

Analyte **17**
Hex:2-PrOH (80:20 *v/v)*

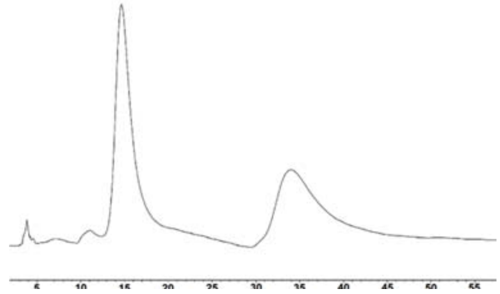

Analyte **17**
100 % EtOH

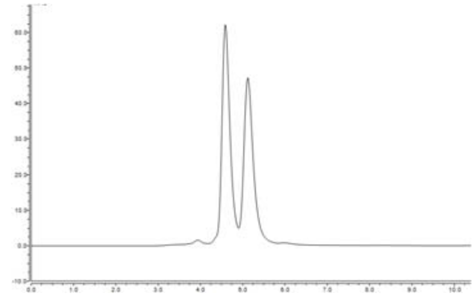

Analyte **17**
100 % 2-PrOH

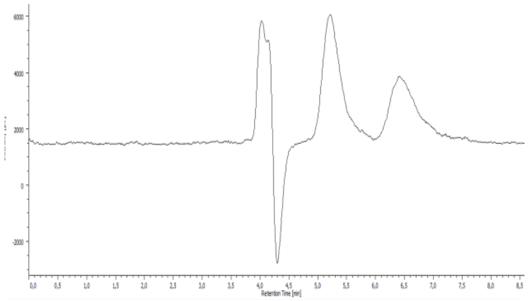

Analyte **17**
MeOH:TEAA pH 4.2 (30:70 *v/v)*

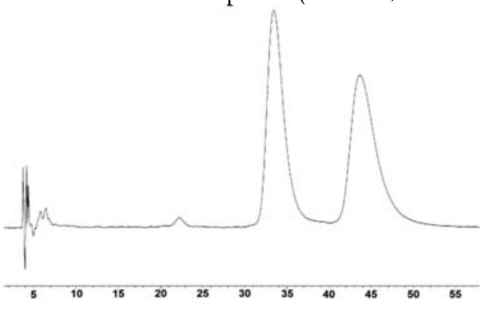

SFigure 3: Chromatograms for the enantioseparation of analytes **15-17** on Chirobiotic T column using different mobile phases. Chromatographic conditions: Flow rate: 0.5 mL/min, UV detection at 254 nm.

Analyte **18**
Hex:EtOH (90:10 *v/v)*

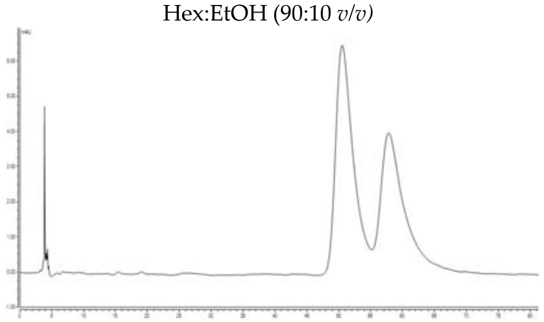

Analyte **19**
Hex:EtOH (90:10 *v/v)*

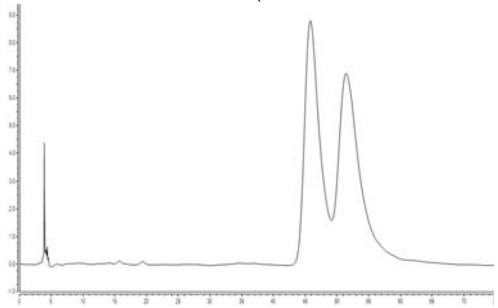

Analyte **19**
Hex:2-PrOH (50:50 *v/v*)

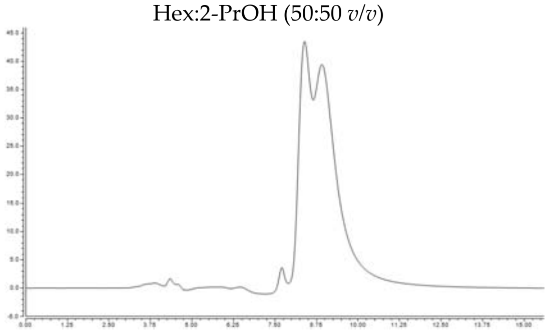

Analyte **19**
MeOH:TEAA pH 4.2 (30:70 *v/v)*

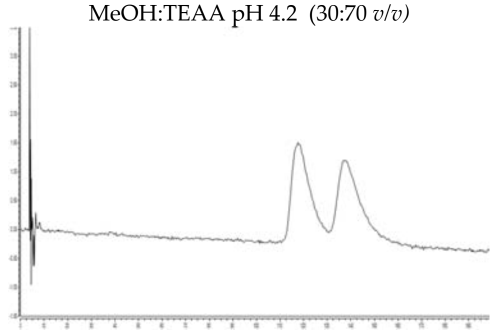

Analyte **20**
Hex:EtOH (80:20 *v/v*)

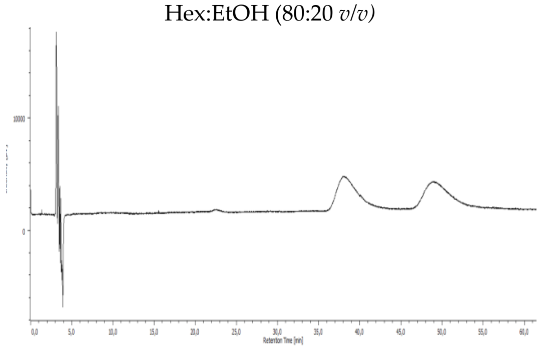

Analyte **20**
Hex:2-PrOH (50:50 *v/v*)

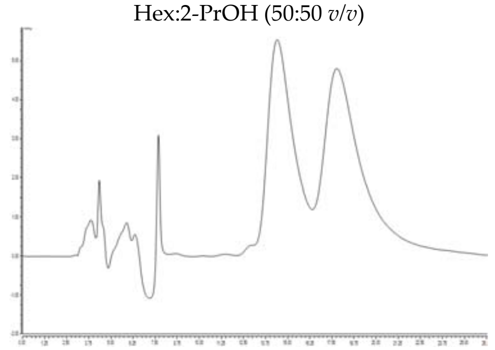

Analyte **20**
100 % EtOH

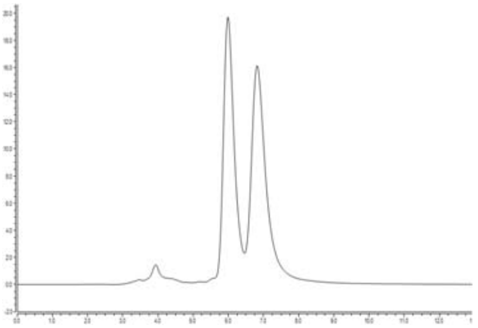

Analyte **22**
Hex:EtOH (80:20 *v/v)*

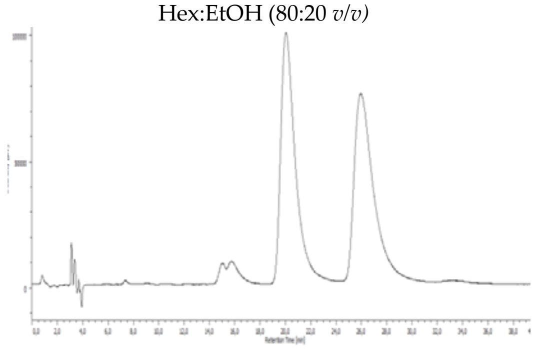

SFigure 4: Chromatograms for the enantioseparation of analytes **18-20** and **22** on Chirobiotic T column using different mobile phases. Chromatographic conditions: Flow rate: 0.5 mL/min, UV detection at 254 nm.

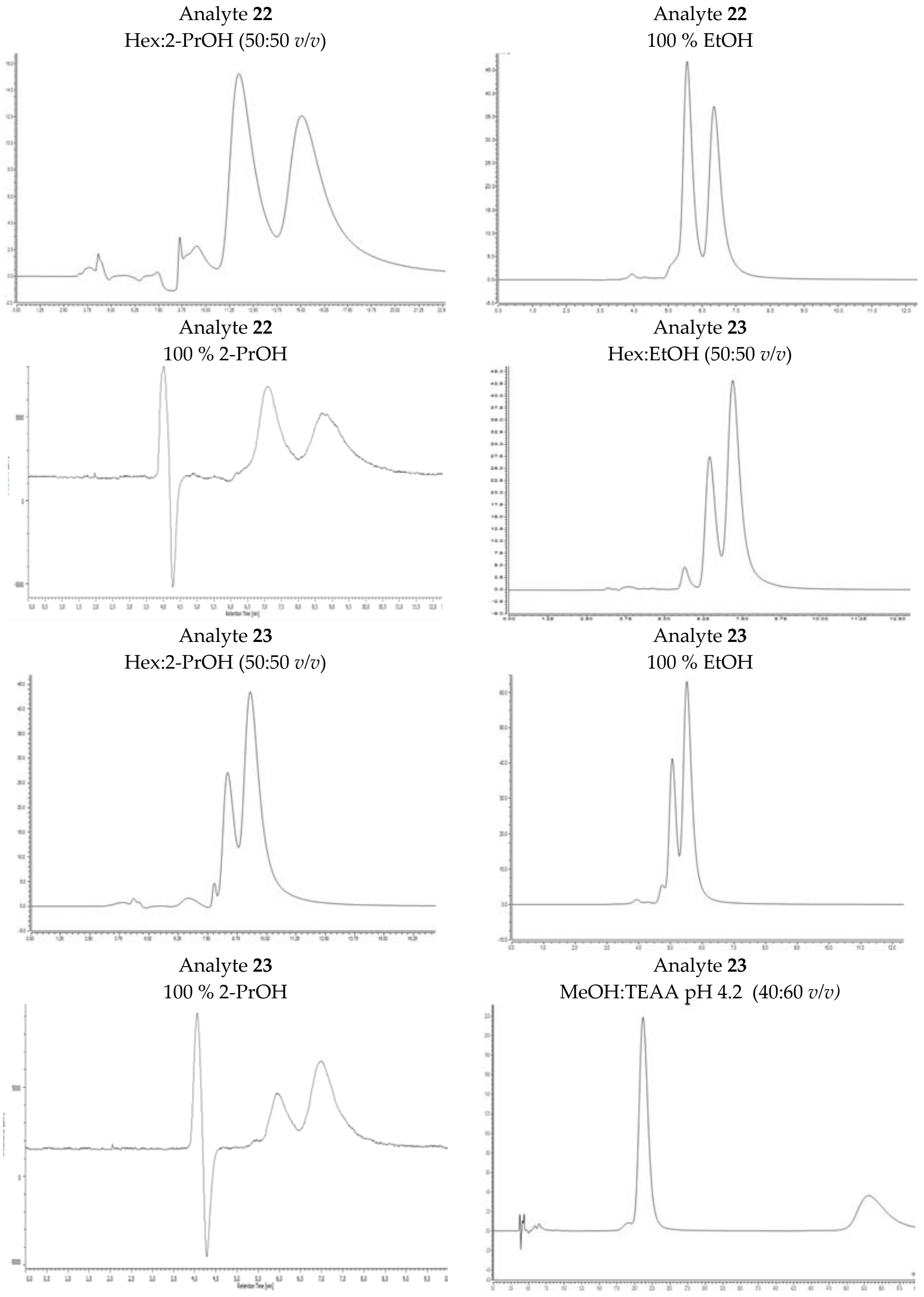

SFigure 5: Chromatograms for the enantioseparation of analytes **22** and **23** on Chirobiotic T column using different mobile phases. Chromatographic conditions: Flow rate: 0.5 mL/min, UV detection at 254 nm.

Analyte **24**
Hex:EtOH (90:10 *v/v)*

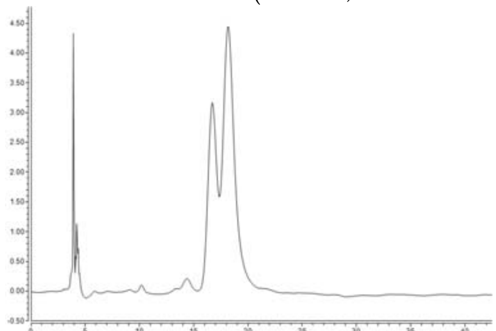

Analyte **25**
Hex:EtOH (90:10 *v/v)*

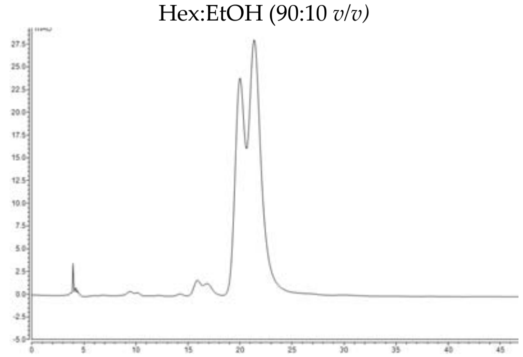

Analyte **26**
Hex:EtOH (90:10 *v/v*)

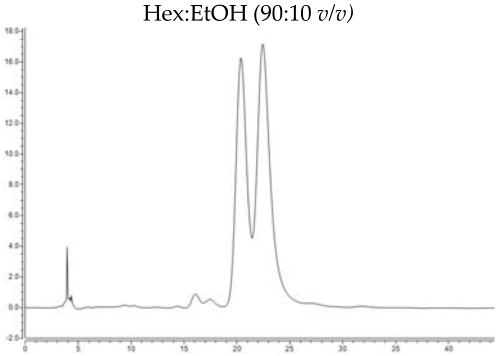

Analyte **26**
MeOH:TEAA pH 4.2 (30:70 *v/v*)

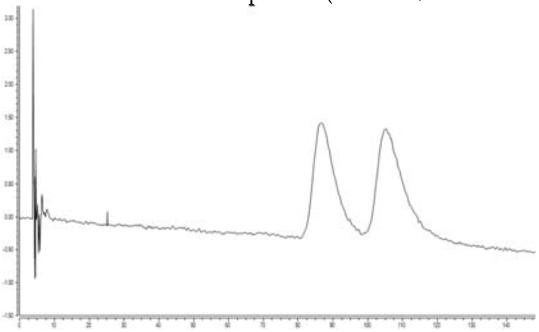

Analyte **27**
MeOH:TEAA pH 4.2 (40:60 *v/v*)

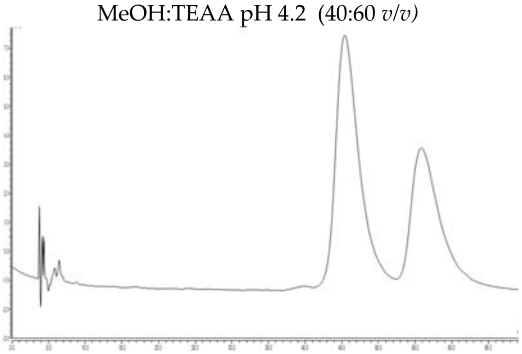

Analyte **28**
Hex:EtOH (90:10 *v/v*)

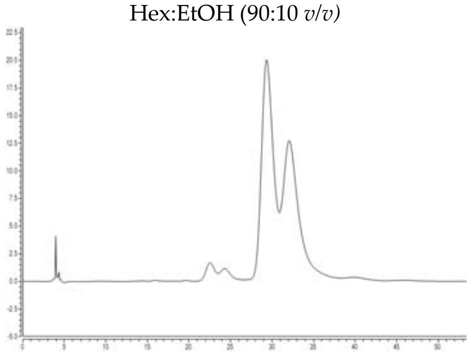

Analyte **29**
Hex:EtOH (90:10 *v/v*)

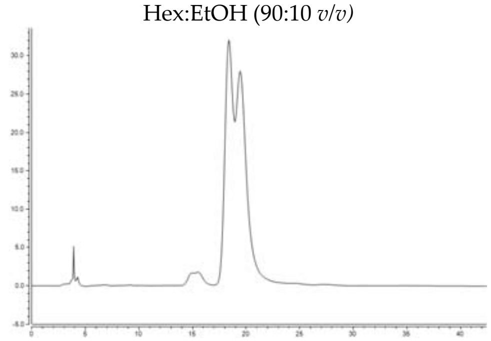

Analyte **30**
Hex:EtOH (90:10 *v/v*)

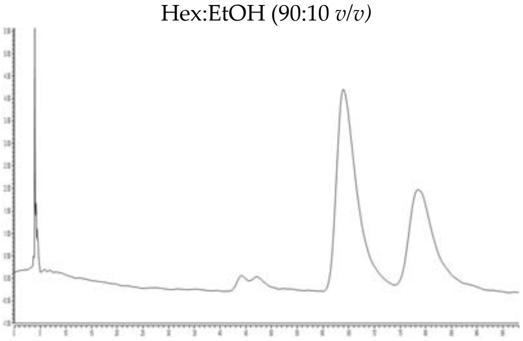

SFigure 6: Chromatograms for the enantioseparation of analytes **24-30** on Chirobiotic T column using different mobile phases. Chromatographic conditions: Flow rate: 0.5 mL/min, UV detection at 254 nm.

Analyte **30**
100 % EtOH

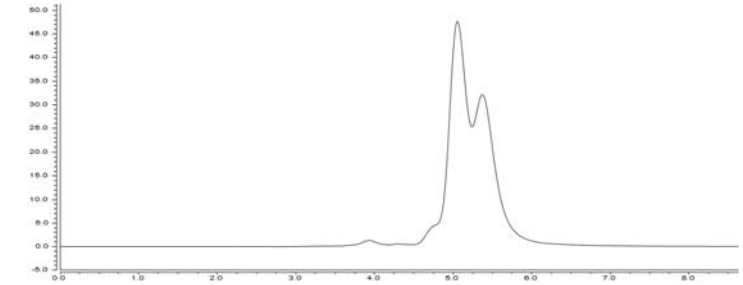

Analyte **30**
100 % 2-PrOH

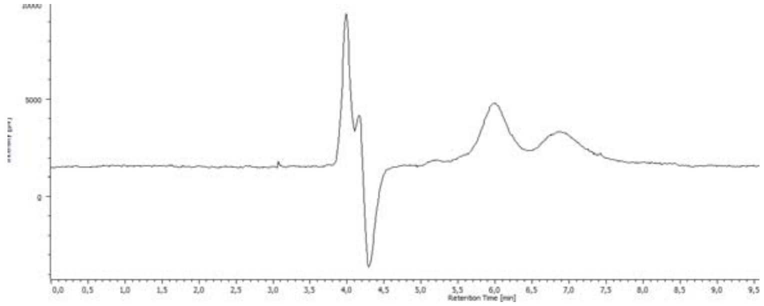

SFigure 7: Chromatograms for the enantioseparation of analytes **30** on Chirobiotic T column using different mobile phases. Chromatographic conditions: Flow rate: 0.5 mL/min, UV detection at 254 nm.

Analyte **1**
100 % MeOH

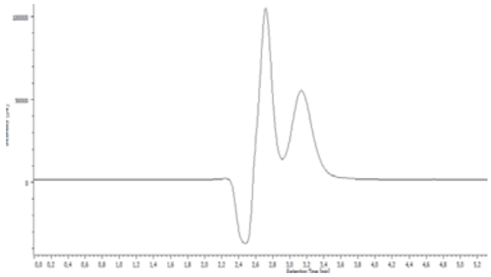

Analyte 2
MeOH:AcOH:TEA (100:0.01:1 *v*/*v*/ *v*)

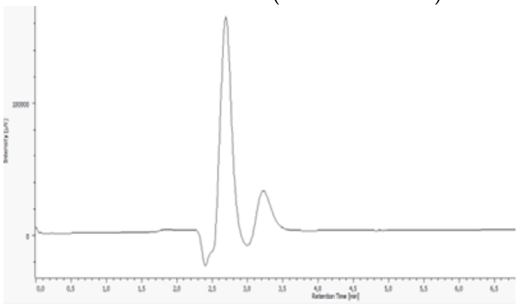

Analyte **2**
100 % MeOH

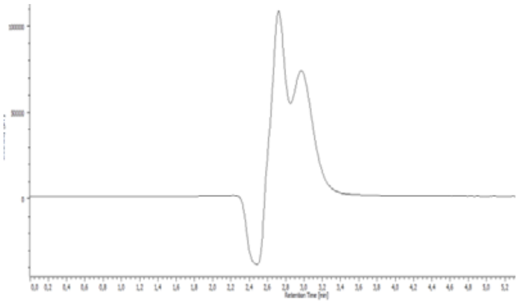

Analyte 2
MeOH:AcOH:TEA (100:0.01:0.01 *v*/*v*/ *v*)

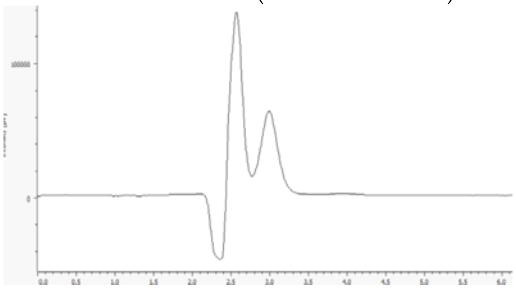

Analyte **4**
100 % MeOH

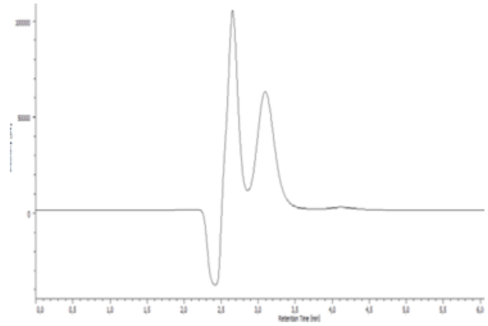

Analyte **4**
MeOH:AcOH:TEA (100:0.1:1 *v*/*v*/ *v*)

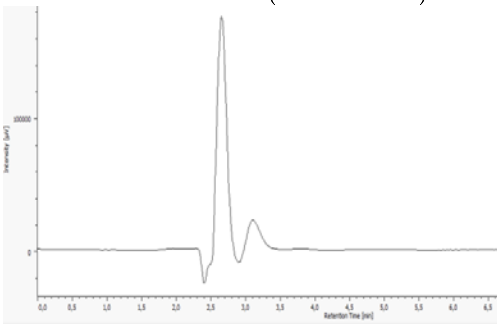

Analyte **5**
100 % MeOH

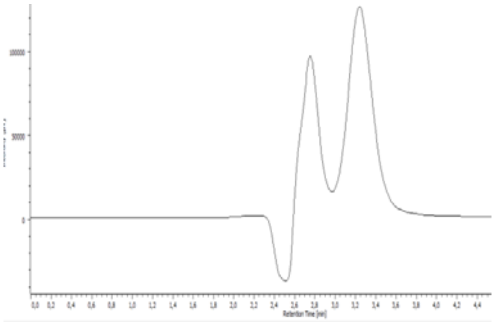

Analyte **9**
MeOH:AcOH:TEA (100:0.1:0.1 *v*/*v*/ *v*)

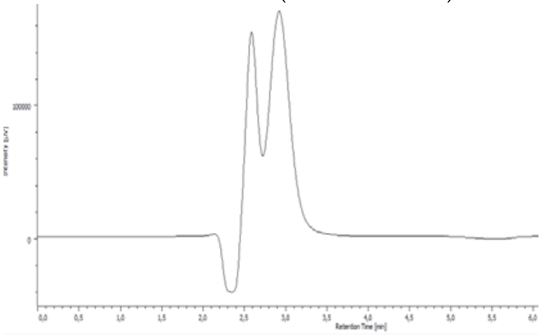

SFigure 8: Chromatograms for the enantioseparation of analytes **1,2,4,5** and **9** on Chirobiotic R column using different mobile phases. Chromatographic conditions: Flow rate: 0.2 mL/min, UV detection at 254 nm.

Analyte **10**
Hex:EtOH (90:10 *v/v)*

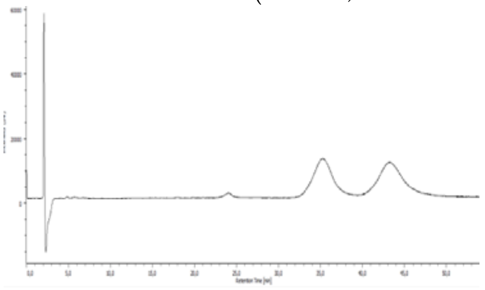

Analyte **11**
Hex:EtOH (90:10 *v/v)*

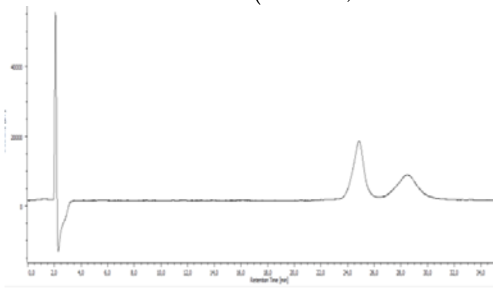

Analyte **12**
Hex:EtOH (90:10 *v/v)*

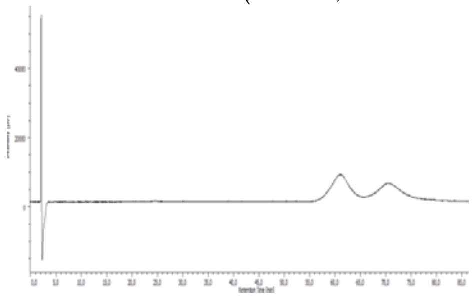

Analyte **13**
MeOH:AcOH:TEA (100:0.1:0.01 *v/v/ v*)

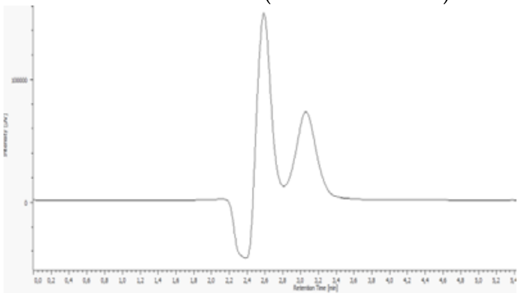

Analyte **17**
Hex:EtOH (90:10 *v/v)*

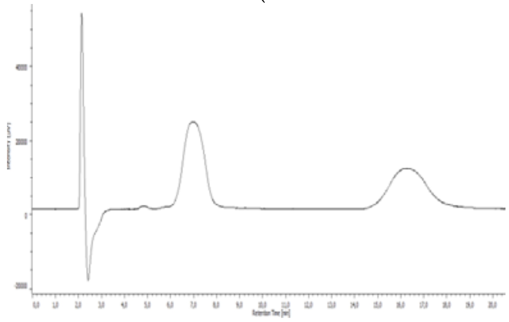

Analyte **20**
Hex:EtOH (90:10 *v/v)*

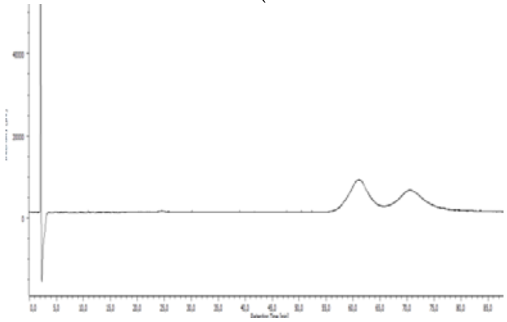

Analyte **20**
MeOH:AcOH:TEA (100:0.1:1 *v/v/ v*)

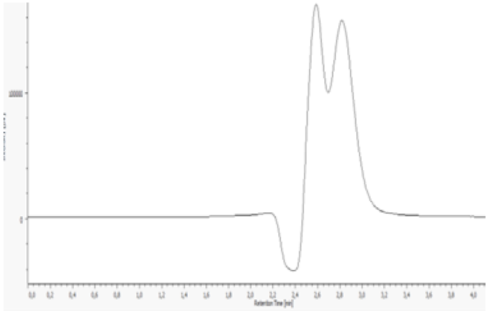

Analyte **22**
Hex:EtOH (90:10 *v/v)*

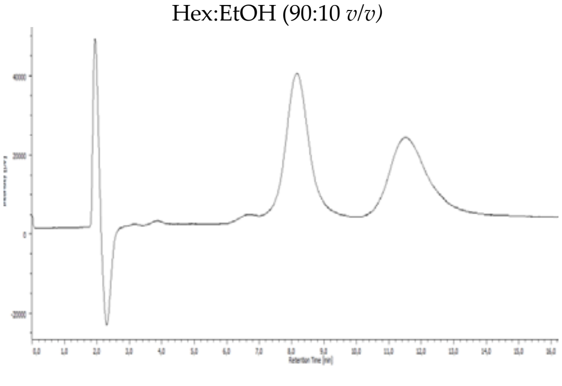

SFigure 9: Chromatograms for the enantioseparation of analytes **10-13, 17, 20** and **22** on Chirobiotic R column using different mobile phases. Chromatographic conditions: Flow rate: 0.2 mL/min, UV detection at 254 nm.

Analyte **10**
Hex:EtOH (80:20 *v/v*)

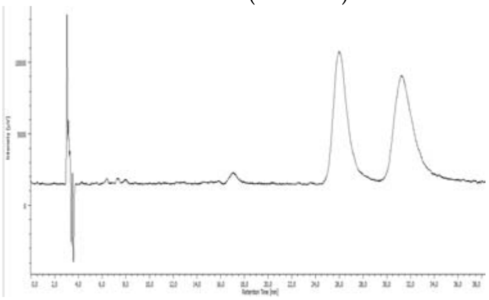

Analyte **11**
Hex:EtOH (80:20 *v/v*)

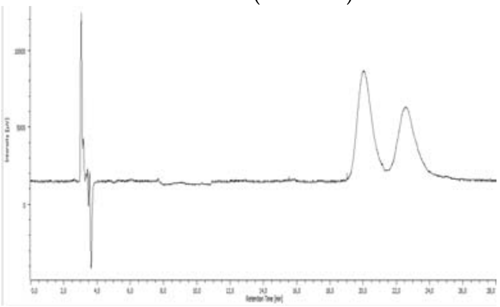

Analyte **12**
Hex:EtOH (80:20 *v/v*)

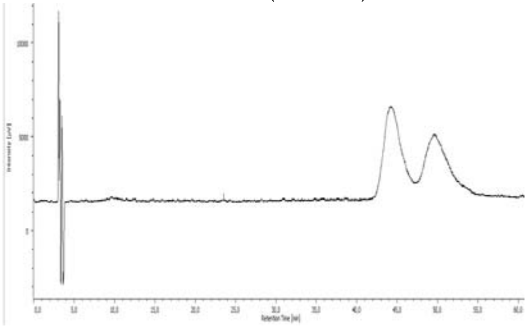

Analyte **14**
MeOH:NH_4OAc pH 6 (50:50 *v/v)*

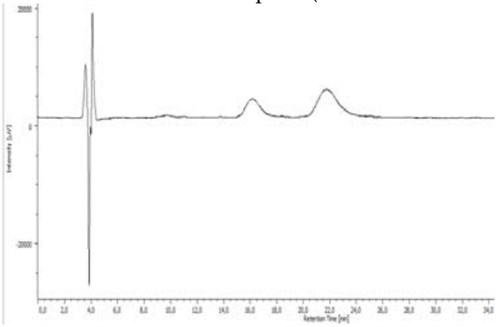

Analyte **14**
MeOH: NH_4OAc pH 4 (50:50 *v/v)*

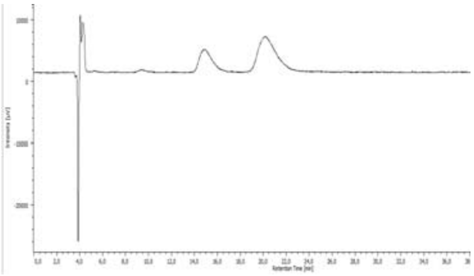

Analyte **15**
MeOH: NH_4OAc pH 6 (50:50 *v/v)*

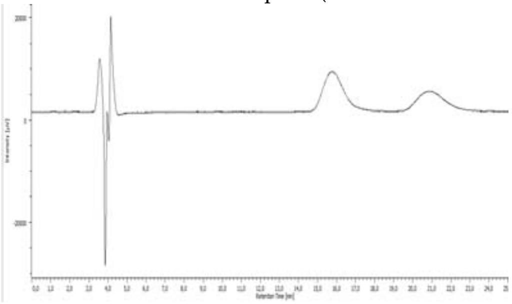

Analyte **15**
MeOH: NH_4OAc pH 4 (50:50 *v/v)*

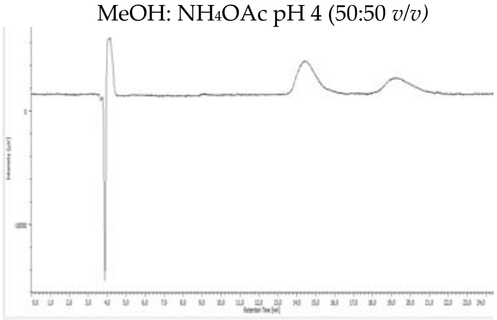

Analyte **16**
MeOH: NH_4OAc pH 6 (50:50 *v/v)*

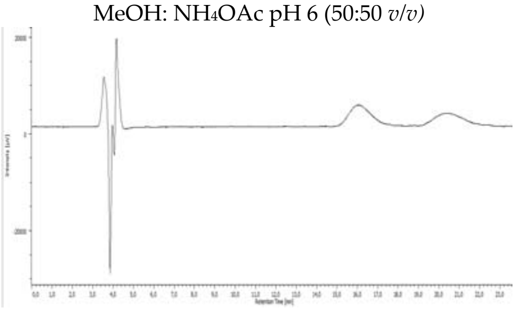

SFigure 10: Chromatograms for the enantioseparation of analytes **10, 11, 12, 14, 15** and **16** on Chirobiotic V column using different mobile phases. Chromatographic conditions: Flow rate: 0.5 mL/min, UV detection at 254 nm.

Analyte **17**
Hex:EtOH (80:20 *v/v*)

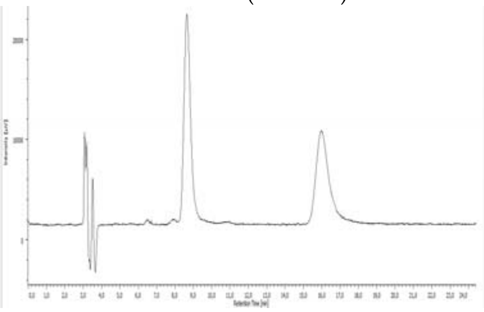

Analyte **17**
100 % EtOH

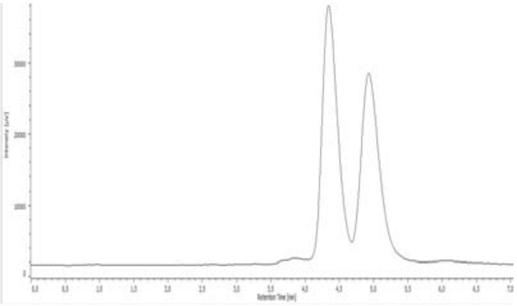

Analyte **17**
100 %2-PrOH

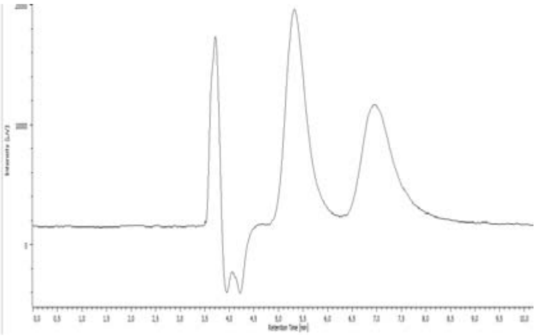

Analyte **17**
MeOH: NH_4OAc pH 6 (50:50 *v/v)*

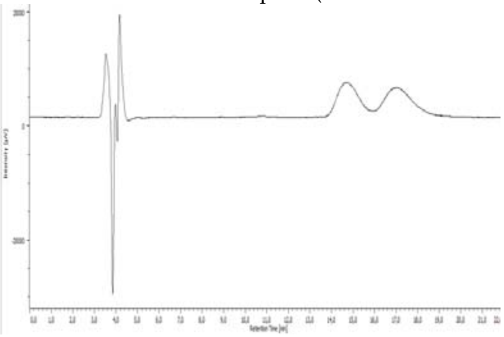

Analyte **17**
MeOH: NH_4OAc pH 4 (50:50 *v/v)*

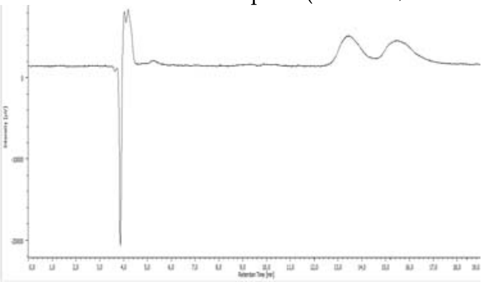

Analyte **18**
MeOH: NH_4OAc pH 6 (50:50 *v/v)*

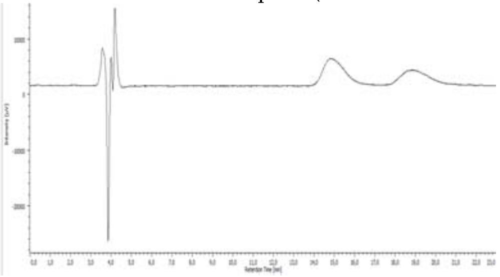

Analyte **18**
MeOH: NH_4OAc pH 4 (50:50 *v/v)*

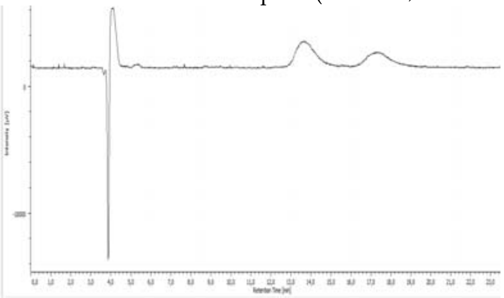

Analyte **19**
MeOH: NH_4OAc pH 6 (50:50 *v/v)*

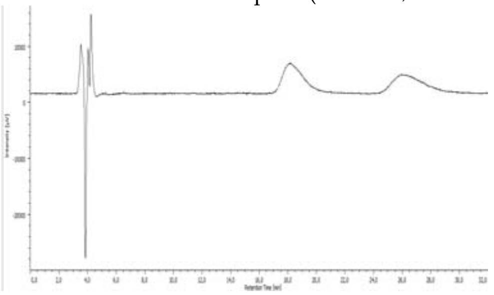

SFigure 11: Chromatograms for the enantioseparation of analytes **17-19** on Chirobiotic V column using different mobile phases. Chromatographic conditions: Flow rate: 0.5 mL/min, UV detection at 254 nm.

Analyte **19**
MeOH: NH_4OAc pH 4 (50:50 *v/v)*

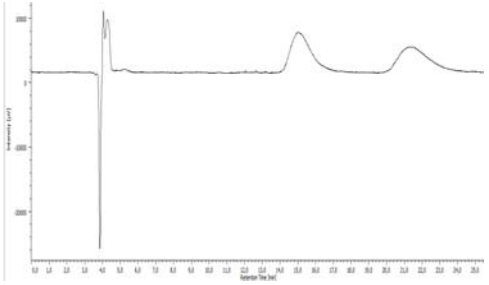

Analyte **20**
Hex:EtOH (80:20 *v/v*)

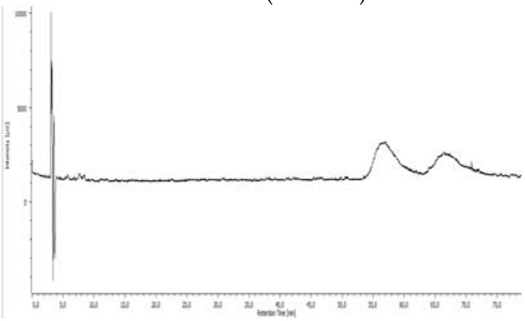

Analyte **22**
MeOH: NH_4OAc pH 6 (50:50 *v/v)*

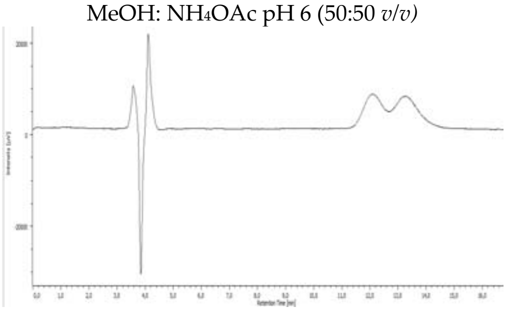

Analyte **22**
MeOH: NH_4OAc pH 4 (50:50 *v/v)*

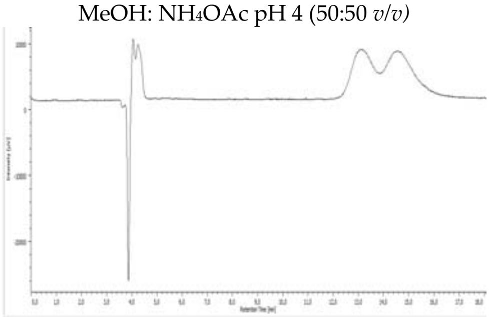

Analyte **23**
Hex:EtOH (80:20 *v/v*)

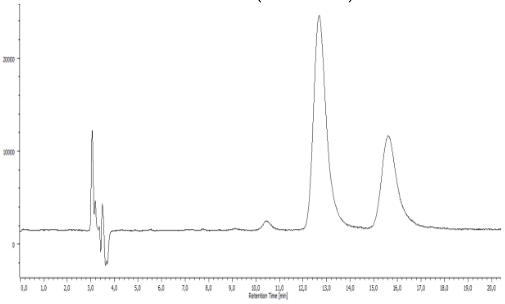

Analyte **23**
MeOH: NH_4OAc pH 6 (50:50 *v/v)*

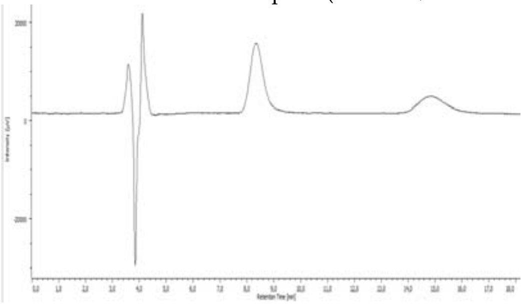

Analyte **23**
MeOH: NH_4OAc pH 4 (50:50 *v/v)*

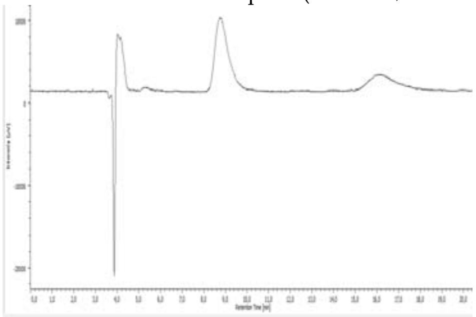

Analyte **25**
MeOH: NH_4OAc pH 6 (50:50 *v/v)*

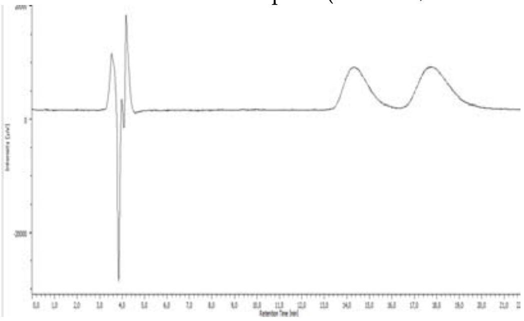

SFigure 12: Chromatograms for the enantioseparation of analytes **19, 20, 22, 23** and **25** on Chirobiotic V column using different mobile phases. Chromatographic conditions: Flow rate: 0.5 mL/min, UV detection at 254 nm.

Analyte **26**
MeOH: NH_4OAc pH 6 (50:50 *v/v)*

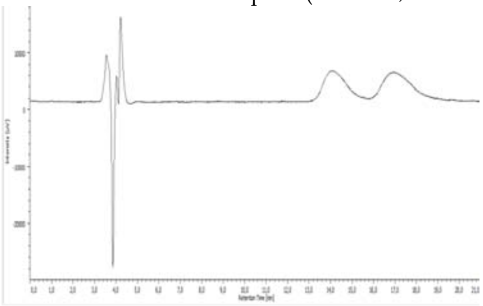

Analyte **26**
MeOH: NH_4OAc pH 4 (50:50 *v/v)*

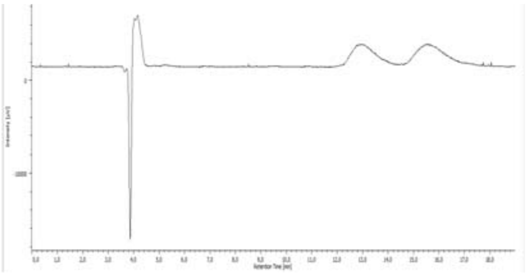

Analyte **27**
MeOH: NH_4OAc pH 6 (50:50 *v/v)*

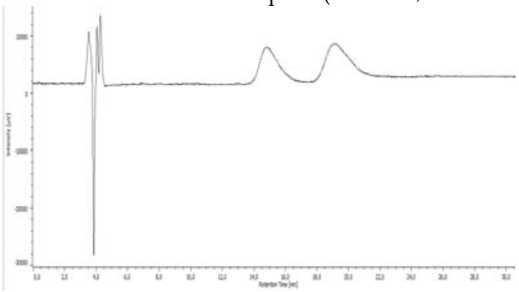

Analyte **27**
MeOH: NH_4OAc pH 4 (50:50 *v/v)*

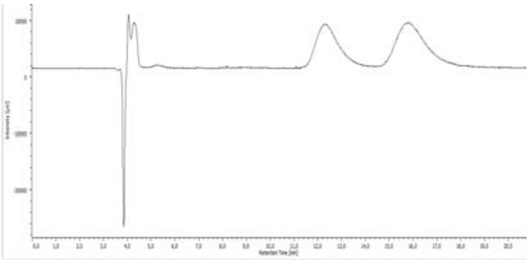

Analyte **28**
MeOH: NH_4OAc pH 6 (50:50 *v/v)*

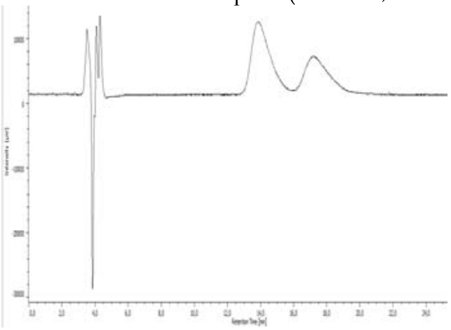

Analyte **28**
MeOH: NH_4OAc pH 4 (50:50 *v/v)*

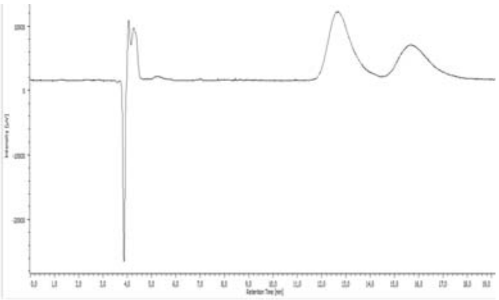

Analyte **29**
MeOH: NH_4OAc pH 6 (50:50 *v/v)*

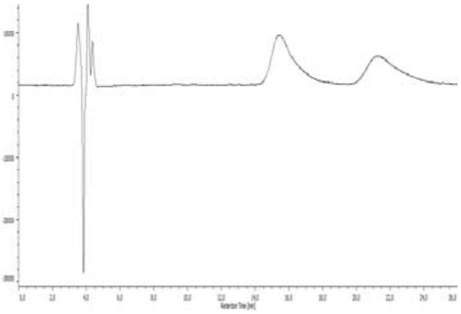

Analyte **29**
MeOH: NH_4OAc pH 4 (50:50 *v/v)*

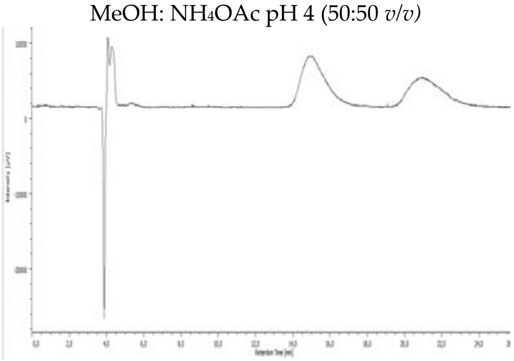

SFigure 13: Chromatograms for the enantioseparation of analytes **26-29** on Chirobiotic V column using different mobile phases. Chromatographic conditions: Flow rate: 0.5 mL/min, UV detection at 254 nm.

Analyte **2**
100 % EtOH

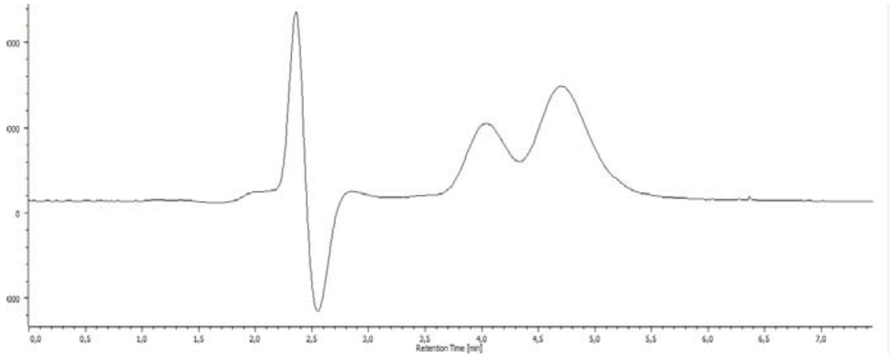

Analyte **17**
Hex:EtOH (70:30 *v*/*v*)

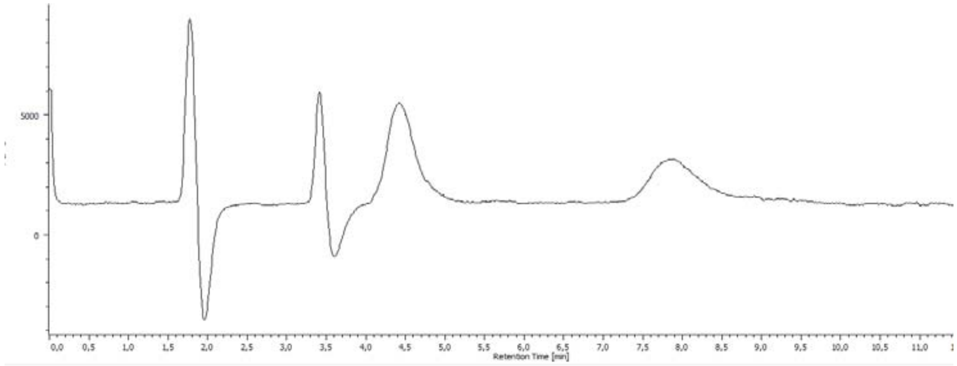

SFigure 14: Chromatograms for the enantioseparation of analytes **2** and **17** on Chirobiotic TAG column using different mobile phases. Chromatographic conditions: Flow rate: 0.2 mL/min, UV detection at 254 nm.
